# Biodegradation of 7-Hydroxycoumarin in *Pseudomonas mandelii* 7HK4 via *ipso*-Hydroxylation of 3-(2,4-Dihydroxyphenyl)-propionic Acid

**DOI:** 10.3390/molecules23102613

**Published:** 2018-10-12

**Authors:** Arūnas Krikštaponis, Rolandas Meškys

**Affiliations:** Department of Molecular Microbiology and Biotechnology, Institute of Biochemistry, Life Sciences Center, Vilnius University, Sauletekio al. 7, LT-10257 Vilnius, Lithuania; rolandas.meskys@bchi.vu.lt

**Keywords:** 7-hydroxycoumarin, 3-(2,4-dihydroxyphenyl)-propionic acid, 3-(2,3,5-trihydroxyphenyl)-propionic acid, *ipso*-hydroxylase, *Pseudomonas mandelii*

## Abstract

A gene cluster, denoted as *hcdABC*, required for the degradation of 3-(2,4-dihydroxyphenyl)-propionic acid has been cloned from 7-hydroxycoumarin-degrading *Pseudomonas mandelii* 7HK4 (DSM 107615), and sequenced. Bioinformatic analysis shows that the operon *hcdABC* encodes a flavin-binding hydroxylase (HcdA), an extradiol dioxygenase (HcdB), and a putative hydroxymuconic semialdehyde hydrolase (HcdC). The analysis of the recombinant HcdA activity in vitro confirms that this enzyme belongs to the group of *ipso*-hydroxylases. The activity of the proteins HcdB and HcdC has been analyzed by using recombinant *Escherichia coli* cells. Identification of intermediate metabolites allowed us to confirm the predicted enzyme functions and to reconstruct the catabolic pathway of 3-(2,4-dihydroxyphenyl)-propionic acid. HcdA catalyzes the conversion of 3-(2,4-dihydroxyphenyl)-propionic acid to 3-(2,3,5-trihydroxyphenyl)-propionic acid through an *ipso*-hydroxylation followed by an internal (1,2-C,C)-shift of the alkyl moiety. Then, in the presence of HcdB, a subsequent oxidative *meta*-cleavage of the aromatic ring occurs, resulting in the corresponding linear product (*2E*,*4E*)-2,4-dihydroxy-6-oxonona-2,4-dienedioic acid. Here, we describe a *Pseudomonas mandelii* strain 7HK4 capable of degrading 7-hydroxycoumarin via 3-(2,4-dihydroxyphenyl)-propionic acid pathway.

## 1. Introduction

The compound 7-hydroxycoumarin (**5**), also known as umbelliferone, is one of the most abundant plant-derived secondary metabolites. It is a parent compound of other, more complex natural furanocoumarins and pyranocoumarins in higher plants [1,2,3]. In the case of plant damage, plants produce a high diversity of natural coumarins as a defense mechanism against insect herbivores as well as fungal and microbial pathogens [4]. For example, simple hydroxycoumarins have antibacterial activity against *Ralstonia solanacearum*, *Escherichia coli*, *Klebsiella pneumoniae*, *Staphylococcus aureus*, and *Pseudomonas aeruginosa* [4,5,6,7,8]. Despite the toxic effects of coumarins, it has been shown that microorganisms evolve to gain the ability to metabolize such compounds.

**Figure 1:**
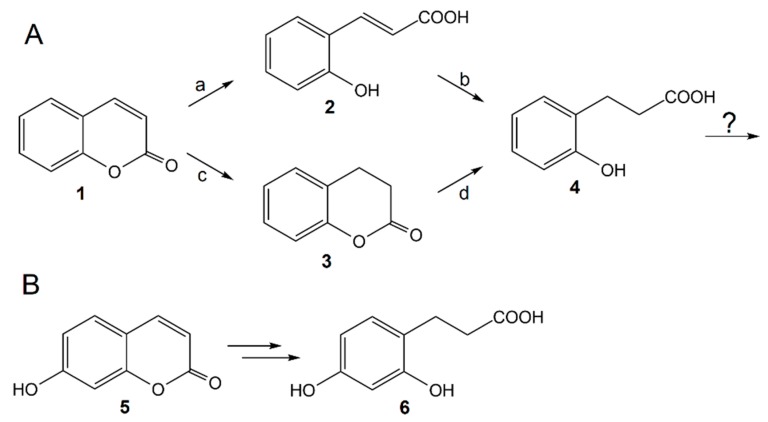
(**A**) Coumarin metabolic routes in *Arthrobacter* spp. (a, b), as well as *Pseudomonas* and *Aspergillus* spp. (c, d). (**B**) Proposed metabolic pathway of 7-hydroxycoumarin in *Pseudomonas* sp. 7HK4 bacteria. **1**—coumarin; **2**—3-(2-hydroxyphenyl)-2-propenoic acid; **3**—dihydrocoumarin; **4**—3-(2-hydroxyphenyl)-propionic acid; **5**—7-hydroxycoumarin; **6**—3-(2,4-dihydroxyphenyl)-propionic acid; **a**—putative coumarin hydrolase; **b**—NADH:*o*-coumarate oxidoreductase; **c**—dihydrocoumarin:NAD[NADP] oxidoreductase; **d**—putative dihydrocoumarin hydrolase.

It has been shown previously that a number of soil microorganisms, such as *Pseudomonas*, *Arthrobacter*, *Aspergillus*, *Penicillium*, and *Fusarium* spp. can grow on coumarin (**1**) as a sole source of carbon [9,10,11,12,13,14,15,16]. The key intermediate during coumarin catabolism in bacteria is 3-(2-hydroxyphenyl)-propionic acid (**4**) [10,11,16]. The bioconversion of coumarin to 3-(2-hydroxyphenyl)-propionic acid can be achieved in two different metabolic pathways, as shown in Figure 1A. Bacteria belonging to the *Arthrobacter* genus enzymatically hydrolyze the lactone moiety to give 3-(2-hydroxyphenyl)-2-propenoic acid (**2**), and then reduce a double bond by using a NADH-dependent enzyme to yield 3-(2-hydroxyphenyl)-propionic acid [11]. In the case of *Pseudomonas* sp. 30-1 and *Aspergillus niger* ATCC 11394 cells, coumarin is initially reduced to dihydrocoumarin (**3**) by a NADH-dependent oxidoreductase and only then enzymatically hydrolyzed [9,10,14]. *Arthrobacter* and *Pseudomonas* species are capable of oxidizing 3-(2-hydroxyphenyl)-propionic acid to 3-(2,3-dihydroxyphenyl)-propionic acid by using specific flavin-binding aromatic hydroxylases [16]. However, no data are available on further conversions of 3-(2,3-dihydroxyphenyl)-propionic acid in these bacteria. Also, no microorganisms or enzymes implicated in the metabolism of any hydroxycoumarin have been identified to date.

Here we describe a *Pseudomonas* sp. 7HK4 strain capable of utilizing 7-hydroxycoumarin as the sole carbon and energy source, and a catabolic pathway of 7-hydroxycoumarin in these bacteria. Analysis of 7-hydroxycoumarin-inducible proteins led to the identification of the genome locus encoding 7-hydroxycoumarin catabolic proteins. The corresponding genes were cloned and heterologously expressed in *E. coli* system. The functions of the recombinant proteins were determined and enzyme activities towards various substrates were evaluated. We show that *Pseudomonas* sp. 7HK4 encodes a distinct 7-hydroxycoumarin metabolic route, which utilizes a flavin monooxygenase responsible for *ipso*-hydroxylation of 3-(2,4-dihydroxyphenyl)-propionic acid (**6**).

## 2. Results

### 2.1. Screening and Identification of 7-Hydroxycoumarin-Degrading Microorganisms

By the means of enrichment culture using various coumarin derivatives, an aerobic strain 7HK4 degrading 7-hydroxycoumarin was isolated from the garden soil in Lithuania (DSMZ accession number DSM 107615). This bacterium was tested for its ability to grow on several coumarin derivatives, such as coumarin, 3-hydroxycoumarin, 4-hydroxycoumarin, 6-hydroxycoumarin, 6-methylcoumarin, 6,7-dihydroxycoumarin, and 7-methylcoumarin, as the sole carbon and energy source in a minimal salt medium. However, of all the aforementioned compounds, the strain 7HK4 was able to utilize 7-hydroxycoumarin only. The strain utilized glucose, which was used as a control substrate in whole-cell reactions. Data on biochemical analysis of this strain are given in the Supplementary Material. The nucleotide sequence of 16S rRNA gene was determined by sequencing of the cloned DNA fragment, which was obtained by PCR amplification. The strain 7HK4 showed the highest 16S rDNA sequence similarity to that from *Pseudomonas* genus and was similar to 16S rDNA from *Pseudomonas mandelii* species according to the phylogenetic analysis, as shown in Figure S1 in the Supplementary Material.

### 2.2. Bioconversion Experiments by Using Whole Cells of Pseudomonas sp. 7HK4

Time course experiments using whole cells of *Pseudomonas* sp. 7HK4 pre-grown in the presence of 7-hydroxycoumarin showed that the cells converted coumarin, 6-hydroxycoumarin, and 6,7-dihydroxycoumarin in addition to 7-hydroxycoumarin, according to the changes in the UV-VIS spectra, as shown in Figure S2 in the Supplementary Material. UV absorption maxima were dropping down over time, although the biotransformation rates of 6-hydroxycoumarin, 6,7-dihydroxycoumarin, and coumarin were approximately 5-fold to 10-fold lower, respectively. After the completion of bioconversions, there were no visible spectra observed for the residual aromatic compound in the reaction mixtures with 7-hydroxycoumarin, except for the reactions with 6-hydroxycoumarin, 6,7-dihydroxycoumarin, and coumarin, which had non-disappearing UV absorption maxima at 260–270 nm wavelengths. These spectra are similar to that of 3-phenylpropionic acid (data not shown), suggesting that 7-hydroxycoumarin-induced 7HK4 strain can only catalyze the hydrolysis and reduction of lactone moiety of coumarin, 6-hydroxycoumarin, and 6,7-dihydroxycoumarin producing 3-(2-hydroxyphenyl)-propionic, 3-(2,5-dihydroxyphenyl)-propionic, and 3-(2,4,5-trihydroxyphenyl)-propionic acids, respectively, comparable to similar biotransformations in other microorganisms [9,10,11,12,13,14,15,16]. In addition, uninduced *Pseudomonas* sp. 7HK4 cells grown in the presence of glucose showed delayed and slower conversion of 7-hydroxycoumarin. The bioconversion process only started 0.5 h after the addition of substrate, suggesting that 7-hydroxycoumarin induced its own metabolism. In addition, uninduced cells did not catalyze any conversion or showed delayed and much slower biotransformations for coumarin, 6,7-dihydroxycoumarin, and 6-hydroxycoumarin, respectively. This demonstrates that in *Pseudomonas* sp. 7HK4 bacteria, the metabolism of 7-hydroxycoumarin is an inducible process.

**Figure 2:**
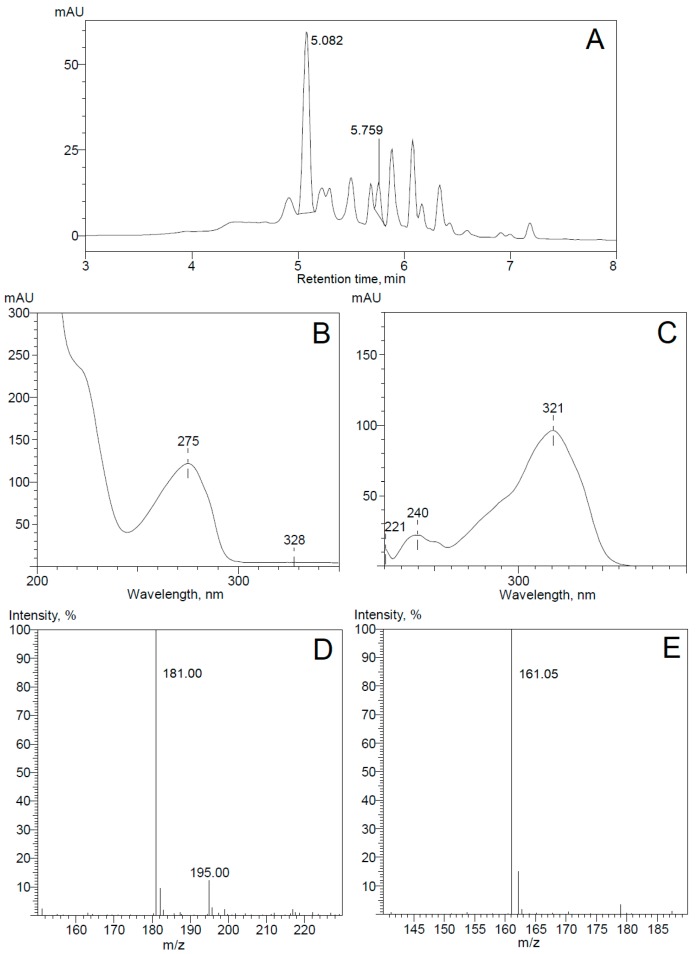
*Pseudomonas* sp. 7HK4 bacteria were grown in the presence of 7-hydroxycoumarin, and produced metabolites were analyzed by HPLC-MS. UV 254 nm trace of metabolites (**A**). UV and MS spectra of peaks with retention times 5.082 min (**B**,**D**) and 5.759 min (**C**,**E**). The negative ions [M−H]^−^ generated are at m/z 181.00 (3-(2,4-dihydroxyphenyl)-propionic acid) and 161.05 (7-hydroxycoumarin).

It has been shown previously that 3-(2-hydroxyphenyl)-2-propenoic and 3-(2-hydroxyphenyl)-propionic acids, as shown in Figure 1B, are the intermediates in a known coumarin metabolic pathway in several microorganisms [9,10,11,14,16]. By analogy, it was suggested that 3-(2,4-dihydroxyphenyl)-propionic acid may be an intermediate metabolite in 7-hydroxycoumarin catabolism, as shown in Figure 1B. The cells of 7HK4 strain pre-cultivated in the presence of 7-hydroxycoumarin catalyzed no conversion of 3-(2-hydroxyphenyl)-2-propenoic or 3-(2-hydroxyphenyl)-propionic acid, however 3-(2,4-dihydroxyphenyl)-propionic acid was straightforwardly consumed. The HPLC-MS analysis of the bioconversion mixtures showed that *Pseudomonas* sp. 7HK4 cells cultivated in the presence of 7-hydroxycoumarin produce 3-(2,4-dihydroxyphenyl)-propionic acid as an intermediate metabolite, as shown in Figure 2. In 7HK4 bacteria grown on glucose, the aforementioned compound was not observed suggesting that 3-(2,4-dihydroxyphenyl)-propionic acid is an intermediate metabolite during 7-hydroxycoumarin degradation.

### 2.3. Identification of 7-Hydroxycoumarin-Inducible Proteins

**Figure 3:**
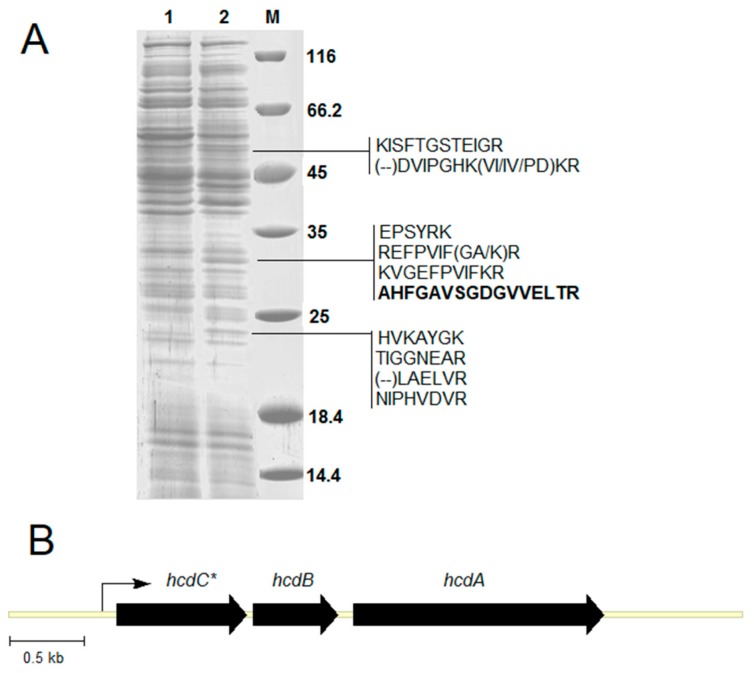
(**A**) SDS-PAGE analysis of cell-free extracts of *Pseudomonas* sp. 7HK4. Bacteria were cultivated in the presence of glucose (lane 1) or 7-hydroxycoumarin (lane 2). M—molecular mass ladder (kDa). The arrows indicate 7-hydroxycoumarin-inducible 23, 32, and 50 kDa proteins. The peptide sequences determined by MS-MS are given on the right. The sequence in bold was identified after the peptide sequences obtained by MS-MS were compared with the partially sequenced genome of *Pseudomonas* sp. 7HK4. (**B**) Organization of *hcd* genes in *Pseudomonas* sp. 7HK4 bacteria. The arrows indicate open reading frames (ORFs) encoding HcdA, HcdB, and HcdC. The gene for the 7-hydroxycoumarin-inducible protein of 31.2 kDa is marked by an asterisk.

To elucidate which enzymes are involved in 7-hydroxycoumarin metabolism, *Pseudomonas* sp. 7HK4 cells were cultivated in a minimal medium supplemented with 7-hydroxycoumarin (0.3 mM) or glucose (0.3 mM) as the sole carbon and energy source. Several 7-hydroxycoumarin-inducible proteins of different molecular mass were observed by using SDS-PAGE analysis of cell-free extracts from *Pseudomonas* sp. 7HK4, as shown in Figure 3A.

Three bands corresponding to the inducible proteins of 23, 32, and 50 kDa, as shown in Figure 3A, were excised from the SDS-PAGE gel and analyzed by MS-MS sequencing. The genome sequence of strain 7HK4 was obtained via Illumina sequencing as described in Materials and Methods. For the identification of corresponding genes of inducible proteins, the sequences of the identified peptides were searched against the partial 7HK4 genome sequence. Thus, the genome fragment was discovered, encoding a 31.2 kDa protein containing 16 aa-long sequence, as shown in the bolded sequence in Figure 3A, identical to that found in 7-hydroxycoumarin-inducible ~32 kDa protein.

### 2.4. Analysis of the Genome Locus Encoding the 7-Hydroxycoumarin-Inducible Protein

Analysis of *Pseudomonas* sp. 7HK4 genome sequences (35 contigs) showed that the inducible 31.2 kDa protein belongs to the fumarylacetoacetate (FAA) hydrolase family, which includes such enzymes as 2-keto-4-pentenoate hydratase, 2-oxohepta-3-ene-1,7-dioic acid hydratase, 2-hydroxy-6-oxo-6-phenylhexa-2,4-dienoate hydrolase, or bifunctional isomerases/decarboxylases (catechol pathway), as shown in Figure S3 in the Supplementary Material. The FAA family proteins are usually involved in the last stages of bacterial metabolism of aromatic compounds [17,18,19], suggesting that 31.2 kDa protein from *Pseudomonas* sp. 7HK4 participates in the final steps of 7-hydroxycoumarin metabolism, after oxidative cleavage of the aromatic ring.

Adjacent to the 31.2 kDa protein-encoding gene, two open reading frames (ORFs) were identified. All three genes are arranged on the same DNA strand, and are separated by short intergenic regions, suggesting that these genes are organized into an operon, as shown in Figure 3B. The putative operon was designated *hcdABC* (**h**ydroxy**c**oumarin **d**egrading operon), where *hcdC* encodes the 31.2 kDa protein. A BLAST analysis of *hcdA* and *hcdB* sequences revealed that these genes encode the putative FAD-binding hydroxylase and ring-cleavage dioxygenase, respectively. HcdA protein was not assigned to any family, but it showed similarity to a putative 2-polyprenyl-6-methoxyphenol hydroxylase, as shown in Figure S3 in the Supplementary Material. This type of enzyme belongs to class A of FAD-binding monooxygenases, which are involved in bacterial degradation of aromatic compounds [20,21,22,23]. The product of the hcdB gene belongs to the cl14632 superfamily that combines a variety of structurally related metalloproteins, including the type I extradiol dioxygenases, as shown in Figure S3 in the Supplementary Material. The type I extradiol dioxygenases catalyze the incorporation of both atoms of molecular oxygen into aromatic substrates that results in the cleavage of the aromatic rings [24,25].

### 2.5. Expression and Substrate Specificity of HcdA Hydroxylase

For further characterization of HcdA hydroxylase, the *hcdA* gene was amplified by PCR and cloned into the pET21b expression vector. The sequence was confirmed by Sanger sequencing. The recombinant C-terminally His_6_-tagged protein was produced in *Escherichia coli* BL21, and purified by affinity chromatography. The purified enzyme migrated as a ~62 kDa band on SDS-PAGE, as shown in Figure S4A in the Supplementary Material, and had a bright yellow color with absorbance maxima at 380 and 450 nm wavelengths, as shown in Figure S4B in the Supplementary Material, suggesting that the protein contains a tightly bound flavin [26,27,28]. The gel-filtration showed that the purified HcdA protein is a monomer, as shown in Figure S4C in the Supplementary Material. The specificity for both flavin and nicotinamide cofactors was investigated. The HcdA hydroxylase was able to utilize either NADH or NADPH, although the oxidation rates of NADPH were almost 2-fold lower. Kinetic characterization of HcdA protein is presented in the Supplementary Material.

The activity of the HcdA enzyme was assayed in the presence of NADH cofactor against various substrates. The highest rate of oxidation of NADH was recorded in the presence of 3-(2,4-dihydroxyphenyl)-propionic acid. A 40-fold lower rate was observed when *trans*-2,4-dihydroxycinnamic acid was used as the substrate. The HcdA was not active towards *trans*-cinnamic, *cis*-2,4-dihydroxycinnamic, 3-(2-hydroxyphenyl)-propionic, 3-(2-hydroxyphenyl)-2-propenoic, 3-(4-hydroxyphenyl)-2-propenoic, 3-(3-hydroxyphenyl)-2-propenoic, 3-(2-bromophenyl)-propionic, 3-(2-nitrophenyl)-propionic, and 3-phenylpropionic acids, cinnamyl alcohol, pyrocatechol, 3-methylcatechol, 4-methylcatechol, 2-propylphenol, 2-propenylphenol, 2-ethylphenol, o-cresol, o-tyrosine, resorcinol, 2,3-dihydroxypyridine, 2-hydroxy-4-aminopyridine, N-methyl-2-pyridone, N-ethyl-2-pyridone, N-propyl-2-pyridone, N-butyl-2-pyridone, indoline, and indole. These data demonstrate that the HcdA is a highly specific monooxygenase, which shows a strong preference to 3-(2,4-dihydroxyphenyl)-propionic acid. The addition of His_6_-tag did not affect the enzymatic activity of the HcdA protein.

**Figure 4:**
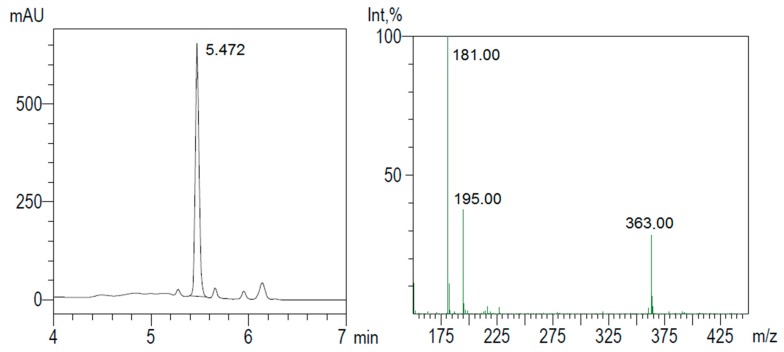
HPLC-MS analysis of 3-(2,4-dihydroxyphenyl)-propionic acid bioconversion mixture in vitro. UV 254 nm trace of 3-(2,4-dihydroxyphenyl)-propionic acid and its hydroxylated product under the same peak with retention time 5.472 min (on the left), and MS spectrum of the dominant peak (on the right). The negative ions [M−H]^−^ generated are at *m*/*z* 181 (3-(2,4-dihydroxyphenyl)-propionic acid), 195 (product of 3-(trihydroxyphenyl)-propionic acid autooxidation), and 363 (dimer of 3-(2,4-dihydroxyphenyl)-propionic acid).

The product of the reaction catalyzed by the HcdA hydroxylase was analyzed by UV-VIS absorption spectroscopy and HPLC-MS. A new UV absorption maximum was observed at 340 and 490 nm upon addition of 3-(2,4-dihydroxyphenyl)-propionic acid to the reaction mixture. The consequent red coloring was observed, which indicated the presence of *para*- or *ortho*-quinone [29], a presumed product of the autooxidation of the corresponding hydroquinone. The same coloration was also observed in vivo when *Pseudomonas* sp. 7HK4 cells were grown in an excess of 7-hydroxycoumarin and when *Escherichia coli* BL21 cells harboring the p4pmPmo plasmid were cultured in the presence of 3-(2,4-dihydroxyphenyl)-propionic acid. HPLC-MS analysis of the reaction mixtures of both in vitro and in vivo bioconversions confirmed the formation of 3-(trihydroxyphenyl)-propionic acid and quinone, found [M−H]^−^ masses were 197.00 (traces seen only in vivo) and 195.00, respectively, as shown in Figure 4. However, the structure of the product was not confirmed at this stage by chemical analysis since it was not possible to chromatographically separate the product from the reaction mixture. The substrate and product have similar structures and chemical properties; therefore, both were detected under the same HPLC-MS chromatogram peak with retention time of ~5.5 min.

### 2.6. Expression and Substrate Specificity of HcdB Dioxygenase

HcdB dioxygenase was expressed from the plasmid pTHPPDO in *E. coli* BL21. All attempts to purify HcdB aerobically resulted in the loss of the enzymatic activity, even in the presence of organic solvents, such as glycerol, ethanol, and acetone that were known as stabilizers for similar enzymes [30,31,32]. The addition of dithiothreitol and ferrous sulfate to the aerobically purified protein did not restore its activity, although it had been shown to activate and/or stabilize other extradiol dioxygenases [30,31,32,33,34].

**Figure 5:**
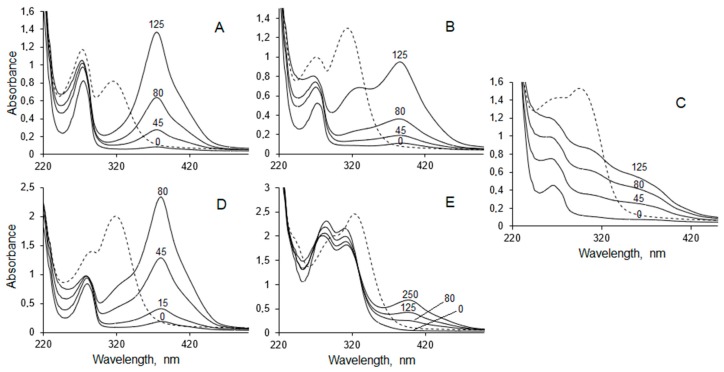
Biotransformations of pyrocatechol (**A**), 3-methylcatechol (**B**), 3-methoxycatechol (**C**), 4-methylcatechol (**D**), and caffeic acid (**E**) by whole cells of *E. coli* BL21 containing *hcdB* gene. Biotransformations were carried out in 50 mM potassium phosphate buffer pH 7.5 (solid line) at 30 °C with 0.5–1 mM of substrate. Incubation time is shown in min. Dashed lines indicate peak shifts to a shorter wavelength after acidification of reaction mixtures.

Therefore, due to the highly unstable nature of the HcdB enzyme, all the activity measurements were carried out in vivo using the whole cells of *E. coli* BL21 transformed with the pTHPPDO plasmid. The bioconversion of pyrocatechol, 3-methylcatechol, 3-methoxycatechol, 4-methylcatechol, and caffeic acids in each case produced yellow products with the absorbance maxima expected for the proximal *meta*-cleavage products of these catechols, as shown by the solid line in Figure 5 [35,36,37]. A weak yellow color was visible in bioconversion mixture containing 3-(2,3-dihydroxyphenyl)-propionic acid, and no changes in color took place, but the absorbance maxima shifts were observed during reactions with pyrogallol, gallacetophenone, 2′,3′-dihydroxy-4′-methoxyaceto-phenone hydrate, 3,4-dihydroxybenzoic acid, 2,3,4-trihydroxybenzoic acid, and 2,3,4-trihydroxy-benzophenone, as shown in Figure S11 in the Supplementary Material. No activity was observed with 1,2,4-benzenetriol and 6,7-dihydroxycoumarin. The *E. coli* cells without the *hcdB* gene showed no activity towards the aforementioned compounds. The expected shift of peaks of the UV-VIS spectra of the reaction products to a shorter wavelength were observed after acidification of the reaction mixtures containing pyrocatechol, 3-methylcatechol, 3-methoxycatechol, 4-methylcatechol, and caffeic acid, as shown by the dashed line in Figure 5 [38,39]. These findings revealed that *hcdB* encodes an extradiol dioxygenase, which can utilize a number of differently substituted catechols.

Furthermore, the HcdB dioxygenase was co-expressed with the HcdA hydroxylase in *E. coli* BL21 cells. The activity of those cells towards 3-(2,4-dihydroxyphenyl)-propionic acid was analyzed. No coloration occurred during the incubation of over 72 h, compared to the formation of a reddish bioconversion product by *E. coli* cells containing the *hcdA* gene only. Products of 3-(2,4-dihydroxyphenyl)-propionic acid conversion by HcdA and HcdB were analyzed by HPLC-MS. Ions with masses 181.00, 195.00, 197.00 ([M−H]^−^) were not detected showing a complete conversion of substrate and its hydroxylated forms. Also, none of the expected ions ([M−H]^−^ 229.00 or [M+H]^+^ 231.00) were observed for a presumed product of the oxidative cleavage of 3-(trihydroxyphenyl)-propionic acid.

### 2.7. Isolation and Identification of 3-(2,4-Dihydroxyphenyl)-propionic Acid Bioconversion Product

**Figure 6:**
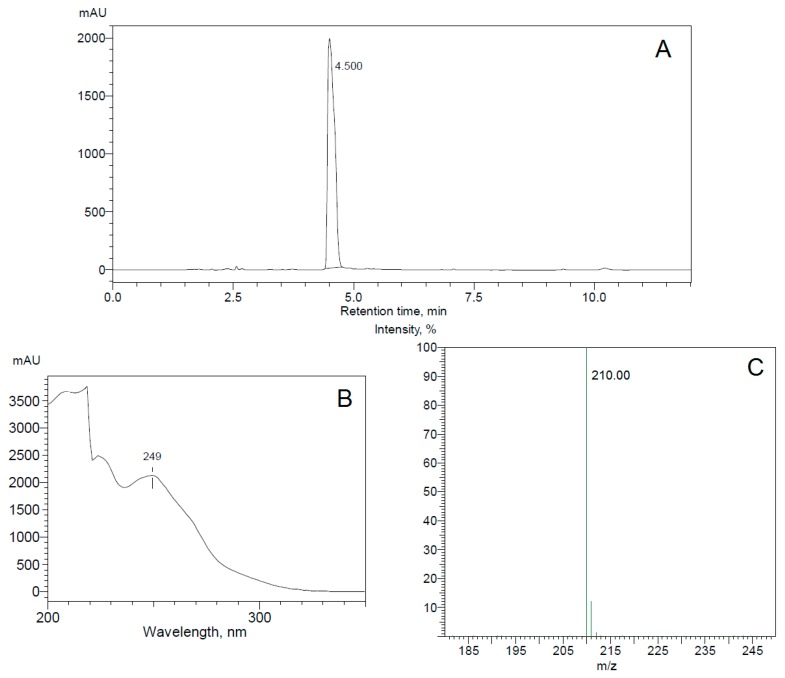
HPLC-MS analysis of 3-(2,4-dihydroxyphenyl)-propionic acid bioconversion mixture in vivo. UV 254 nm trace of picolinic acid derivative with retention time of 4.500 min (**A**), UV spectrum (**B**) and MS spectrum (**C**) of the dominant peak. The negative ions [M−H]^−^ generated are at *m*/*z* 210.00.

Due to difficulties in detecting colorless *meta*-cleavage product of catechol derivative, and since no reasonable mass spectra could be registered, we decided to transform a cleavage product into the derivative of picolinic acid by incubation with NH_4_Cl as described in Materials and Methods. The formation of a picolinic acid derivative was proven by HPLC-MS analysis, which showed the formation of [M−H]^−^ ion 210.00 mass, as shown in Figure 6, corresponding to the addition of NH_3_ to the *meta*-cleavage product and the loss of two H_2_O molecules [40].

**Figure 7:**
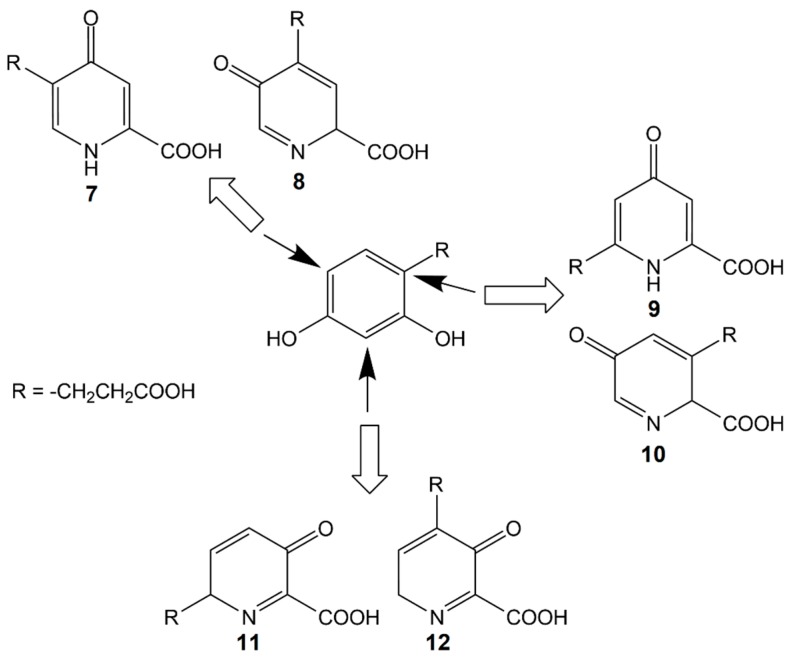
Suggested structures of *oxo*-picolinic acid derivative, formed during oxidative ring cleavage of 3-(2,4-dihydroxyphenyl)-propionic acid and conversion of the ring fission product. Solid lines indicate possible positions of hydroxylation by HcdA enzyme; hollow arrays indicate the probable *oxo*-picolinic acid derivatives forming after hydroxylation at each position.

**Scheme 1:**
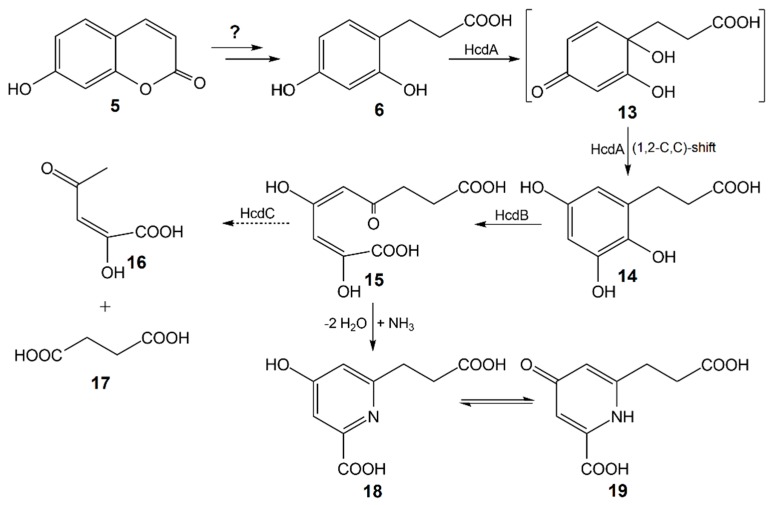
The proposed metabolic pathway of 7-hydroxycoumarin in *Pseudomonas* sp. 7HK4 cells. Incubation of the compound **5** with NH_4_Cl gives picolinic acid derivative. **5**—7-hydroxycoumarin; **6**—3-(2,4-dihydroxyphenyl)-propionic acid; **13**—3-(1,2-dihydroxy-4-oxocyclohexa-2,5-dienyl)-propanoic acid; **14**—3-(2,3,5-trihydroxyphenyl)-propionic acid; **15**—(*2E*,*4E*)-2,4-dihydroxy-6-oxonona-2,4-dienedioic acid; **16**—(*E*)-2-hydroxy-4-oxopent-2-enoic acid; **17**—succinic acid; **18**—6-(2-carboxyethyl)-4-hydroxypicolinic acid; **19**—6-(2-carboxyethyl)-4-oxo-1,4-dihydropyridine-2-carboxylic acid; HcdA—3-(2,4-dihydroxyphenyl)-propionic acid 1-monooxygenase; HcdB—3-(2,3,5-trihydroxyphenyl)-propionic acid 1,2-dioxygenase; HcdC—putative (*2E*,*4E*)-2,4-dihydroxy-6-oxonona-2,4-dienedioic acid hydrolase. The dashed arrow indicates a hypothetical reaction.

The ^1^H NMR spectrum of this derivative [δ 7.73 (s, 1H), 6.89 (s, 1H), 2.65 (t, *J* = 7.4 Hz, 2H), 2.38 (t, *J* = 7.4 Hz, 2H)] showed a set of two aryl protons that, from the coupling pattern (singlet + singlet), were in *meta*- or *para*-positions to each other on the aromatic ring [41], as shown in Figure S12 in the Supplementary Material. The appearance in the spectrum of two triplets with chemical shifts of 2.38 and 2.65 ppm indicated the presence of four methylene protons [41]. The ^13^C NMR spectrum [δ 181.51, 179.99, 171.01, 143.14, 136.74, 130.11, 115.22, 35.50, 24.00] showed two sp^3^ carbons with chemical shifts of 24.00 and 35.50 ppm, and three sp^2^ carbons of carbonyl groups with chemical shifts of 171.01, 179.99, and 181.51 ppm, respectively, as shown in Figure S13 in the Supplementary Material. The carbonyl carbon atoms were the most strongly deshielded and their resonances formed a separate region at the highest frequency. Another four sp^2^ carbon signals were in the aromatic carbon region [41,42]. The presence of the third carbonyl group indicated the formation of *oxo*-pyridine, for which six possible theoretical structures of *oxo*-picolinic acid derivative were presumed, as shown in Figure 7. Since the ^1^H NMR spectrum showed a set of two singlet aryl protons in *meta*- or *para*-positions to each other, only structures **7** and **9**, as shown in Figure 7, were further analyzed. Besides, pyridine aromatic carbons are usually differentiated into two resonances at higher field (C-3/5, *meta* position) and three at lower field (C-2/6, *ortho* position; C-4, *para* position), where the electron-withdrawing effect of nitrogen is effective [41,42]. The chemical shift of 115.22 ppm showed that the analyzed compound had relatively strongly shielded unsubstituted aromatic carbon, which should be in *meta*-position from nitrogen, in *ortho*-position from the carbonyl group, and in *meta*- or *para*-position from the carboxyl group (C-5 atom), as shown in Figure S14 in the Supplementary Material [43,44]. This led to the conclusion that structure **9**, as shown in Figure 7, 6-(2-carboxyethyl)-4-oxo-1,4-dihydropyridine-2-carboxylic acid, was formed during incubation of *meta*-cleavage product of catechol derivative with NH_4_Cl, as depicted in Scheme 1. These data allowed the reconstruction of the consecutive oxidation of 3-(2,4-dihydroxyphenyl)-propionic acid catalyzed by the HcdA and HcdB enzymes. Hence, 3-(2,3,5-trihydroxyphenyl)-propionic acid (**14**) was the product of oxidation of 3-(2,4-dihydroxyphenyl)-propionic acid by the HcdA hydroxylase. The molecular mass of 198.17 of 3-(2,3,5-trihydroxyphenyl)-propionic acid and capability to form *para*-quinone agreed with the UV-VIS and HPLC-MS data on the bioconversion of 3-(2,4-dihydroxyphenyl)-propionic by the HcdA hydroxylase. The formation of 3-(2,3,5-trihydroxyphenyl)-propionic acid from 3-(2,4-dihydroxyphenyl)-propionic acid was possible only through oxidative *ipso*-rearrangement, a unique reaction where *ipso*-hydroxylation (**13**) of the 3-(2,4-dihydroxyphenyl)-propionic acid takes place with a simultaneous shift of the propionic acid group to the vicinal position, as shown in Scheme 1 [45,46,47,48]. During the second step, 3-(2,3,5-trihydroxyphenyl)-propionic acid was cleaved by HcdB extradiol dioxygenase at the *meta*-position leading to the formation of (*2E*,*4E*)-2,4-dihydroxy-6-oxonona-2,4-dienedioic acid (**15**). The further imine formation and tautomerization in the presence of ammonium ions [38,49] led to 6-(2-carboxyethyl)-4-*oxo*-1,4-dihydropyridine-2-carboxylic acid (**19**), as shown in Scheme 1.

### 2.8. Expression of the HcdC Protein

HcdC was expressed from the plasmid p2K4PH in *E. coli* BL21. The addition of *E. coli* extracts containing the HcdC protein did not cause decolorization of the *meta*-cleavage products of 3-(2,3-dihydroxyphenyl)-propionic acid, pyrocatechol, 3-methylcatechol, or 4-methylcatechol formed in the presence of the HcdB extradiol dioxygenase. Also, the addition of NAD(P)^+^ to these reaction mixtures did not induce decolorization of the *meta*-cleavage products [50]. To confirm the function of HcdC, *E. coli* BL21 cells were transformed with p4pmPmo and pCDF-BC plasmids. The expression of *hcdA*, *hcdB,* and *hcdC* genes in *E. coli* cells was confirmed by SDS-PAGE, the enzymes migrated as 62, 31, and 20 kDa bands, respectively, as shown in Figure S15 in the Supplementary Material.

The bioconversion of 3-(2,4-dihydroxyphenyl)-propionic acid was conducted in *E. coli* cells containing all three recombinant proteins. Later, the reaction mixture was incubated with NH_4_Cl, and the reaction products were analyzed by HPLC-MS. The ions [M−H]^−^ and [M+H]^+^ with masses of 210.00 and 212.00, respectively, were not detected, compared to the bioconversion mixture with *E. coli* cells containing *hcdAB* genes only. This showed a complete conversion of *meta*-cleavage product of the catechol derivative, therefore no picolinic acid derivative could be obtained. No reaction products of (*2E*,*4E*)-2,4-dihydroxy-6-oxonona-2,4-dienedioic acid hydrolysis by the HcdC protein were identified. We suggest that the later compound was converted to succinic acid (**17**), which entered the Krebs cycle, and (*E*)-2-hydroxy-4-oxopent-2-enoic acid (**16**), which could be further converted by *E. coli* cells, thus complicating the extraction of these reaction products. Nevertheless, it may be concluded that all three enzymes encoded by the *hcdABC* operon are responsible for the catabolism of 3-(2,4-dihydroxyphenyl)-propionic acid in *Pseudomonas* sp. 7HK4 bacteria.

## 3. Discussion

Although coumarins are widely abundant in nature and are intensively used in biotechnology as precursory compounds [51], the metabolic pathways in microorganisms are still not known in sufficient detail. In this study, *Pseudomonas* sp. 7HK4 strain was isolated from soil and it was shown that these bacteria can utilize only 7-hydroxycoumarin as a sole source of carbon and energy. Several other coumarin derivatives were also tested, but none of those substrates support the growth of *Pseudomonas* sp. 7HK4. However, the experiments with the 7-hydroxycoumarin-induced whole cells show that *Pseudomonas* sp. 7HK4 has the enzymes that are able to transform coumarin and 6-hydroxycoumarin. The products of these biotransformations give UV spectra similar to the UV spectrum of 3-phenylpropionic acid, suggesting that *Pseudomonas* sp. 7HK4 bacteria can catalyze hydrolysis and reduction of lactone moiety of these substrates. Furthermore, 3-(2,4-dihydroxyphenyl)-propionic acid has been identified as an intermediate metabolite during biotransformations of 7-hydroxycoumarin. This finding agrees with the data published for the conversion of coumarin by *Pseudomonas* spp., *Arthrobacter* spp., and *Aspergillus* spp., which all catalyze the hydrolysis and reduction of coumarin producing 3-(2-hydroxyphenyl)-propionic acid as the main intermediate [9,10,11,14]. However, compared with *P. mandelii* 7HK described in this study, little is known about the bioconversion of other coumarin derivatives as well as further conversions of 3-(2-hydroxyphenyl)-propionic acid in bacteria listed above.

The analysis of the 7-hydroxycoumarin-inducible proteins lead to the identification of the genomic locus *hcdABC* encoding the enzymes required for 3-(2,4-dihydroxyphenyl)-propionic acid degradation. A BLAST search uncovered that *hcdA*, *hcdB*, and *hcdC* encode an aromatic flavin-binding hydroxylase, a ring-cleavage dioxygenase and a hydrolase, respectively. Biochemical analysis of the HcdA protein confirmed its similarity to class A FAD-binding enzymes (FMOs) [20,21,22,23,26,27,28]. HcdA is functionally related to previously well-described hydroxylases, such as OhpB monooxygenase from *Rhodococcus* sp. V49 [52], MhpA monooxygenase from *E. coli* K-12 [53] and *Comamonas testosteroni* TA441 [54], HppA monooxygenase from *Rhodococcus globerulus* PWD1 [31], and also *para*-hydroxybenzoate hydroxylase (PHBH) from *P. fluorescens* [55]. HcdA appears to have a high specificity for 3-(2,4-dihydroxyphenyl)-propionic acid, converting it to 3-(2,3,5-trihydroxyphenyl)-propionic acid, and a 40-fold lower activity towards *trans*-2,4-dihydroxycinnamic acid. Other structurally related substrates are not used by HcdA. Unlike HcdA, other related FMOs have a broader specificity for substrates. For example, OhpB monooxygenase is capable to oxidize 2-hydroxy-, 3-hydroxyphenylpropionic and cinnamic acids [52]. The HppA enzyme is more specific to 3-hydroxyphenylpropionic, but 4-chlorophenoxyacetic as well as 4-methyl-2-chlorophenoxyacetic acids are also oxidized [31]. On the other hand, all described FMOs including HcdA are NAD(P)H dependent, which reduces flavin for the hydroxylation of substrates [20,21,22,23]. The narrow specificity of HcdA to its natural substrate is typical for class A flavoproteins and shows the importance of HcdA enzyme in metabolism of 7-hydroxycoumarin. The kinetic analysis of HcdA yields *K*_m_ values of 50.10 ± 3.50 µM and 13.00 ± 1.20 µM for NADH and 3-(2,4-dihydroxyphenyl)-propionic acid, respectively. However, no kinetic parameters have been reported for OhpB, MhpA, and HppA hydroxylases for comparison, though PHBH has been shown to have a *k*_cat_ of 22.83 s^−1^ [56], which is 3-fold higher than a turnover number for the HcdA enzyme.

The hydroxylated product of the HcdA protein was analyzed by oxidizing it with the HcdB dioxygenase, followed by a chemical modification to the corresponding derivative of picolinic acid. The structure of the later compound was confirmed by ^1^H NMR and ^13^C NMR spectra. This allowed the reconstruction of the reaction products of both HcdA and HcdB enzymes. It was shown that a hydroxylation of 3-(2,4-dihydroxyphenyl)-propionic acid occurs at *ipso*-position of phenolic ring followed by internal rearrangements involving (1,2-C,C)-shift (NIH shift) of propionic acid moiety, hence forming 3-(2,3,5-trihydroxyphenyl)-propionic acid, as shown in Scheme 1. This would explain both the high specificity of HcdA enzyme for substrates with *para*-substituted phenol and inability of *Pseudomonas* sp. 7HK4 bacteria to utilize coumarin derivatives other than 7-hydroxycoumarin as the sole source of carbon and energy. Only a few classes of enzymes are able to catalyze *ipso*-reactions: laccases, peroxidases, dioxygenases, glutathione S-transferases (GST), cytochrome P450-dependent monooxygenases (CYP), and flavin-dependent monooxygenases. Among the known examples of *ipso*-enzymes, there are dioxygenases from *Comamonas testosteroni* T2 and *Sphingomonas* sp. strain RW1, which are involved in the desulfonation of 4-sulfobenzoate by *ipso*-substitution [57,58]. A rat liver CYP system is able to convert *p*-chloro, *p*-bromo, *p*-nitro, *p*-cyano, *p*-hydroxymethyl, *p*-formyl, and *p*-acetyl phenols to hydroquinone by *ipso*-hydroxylation [59]. GST is capable of catalyzing desulfonylation of sulfonylfuropyridine compounds by nucleophilic attack of the glutathione sulfur atom at *ipso*-position [60]. These are the examples of electrophilic or nucleophilic *ipso*-substitution reactions, however in some cases, a primary *ipso*-group is not eliminated, and instead it is shifted to *meta*-position. NIH shift restabilizes cyclohexadienone intermediate, because it leads to a rearomatization [45,46,47,48]. We showed that, similarly to flavin-dependent monooxygenases from *Sphingomonas* sp. TTNP3 and *Sphingobium xenophagum* strains, which are responsible for the degradation of alkylphenols, such as bisphenol A, octylphenol, *t*-butylphenol, *n*-octyloxyphenol, and *t*-butoxyphenol, HcdA-catalyzed reaction involves a NIH shift. Usually, NIH shift products are formed during the side reactions, and these internal rearrangements of an alkyl group upon the *ipso*-hydroxylation are spontaneous and non-enzymatic in *Sphingomonas* sp. strains [47,61]. An interesting novelty is that HcdA hydroxylase produces only one product, which has the *ipso*-group shifted to the *meta*-position. Therefore, we propose that in the case of HcdA, NIH shift occurs enzymatically, but not spontaneously or by a dienone-phenol rearrangement mechanism [62], since all bioconversions were performed under neutral or basic conditions. Although a further investigation is needed to determine the exact mechanism of the HcdA enzyme activity.

The analysis of the extradiol dioxygenase HcdB shows that this enzyme has a lower specificity for substrates. It catalyzes a conversion of the hydroxylated product of the HcdA enzyme to (*2E*,*4E*)-2,4-dihydroxy-6-oxonona-2,4-dienedioic acid, as shown in Scheme 1. Also, HcdB is capable oxidizing pyrocatechol, 3-methylcatechol, 3-methoxycatechol, 4-methylcatechol, 3-(2,3-dihydroxyphenyl)-propionic, and caffeic acids using a *meta*-cleavage mechanism forming yellow products. HcdB belongs to type I, class II extradiol dioxygenases [24,25], and is functionally related to OhpD catechol 2,3-dioxygenase from *Rhodococcus* sp. V49 [52], MhpB extradiol dioxygenase from *E. coli* K-12 [53,63], MpcI extradiol dioxygenase from *Alcaligenes eutrophus* [63], HppB extradiol dioxygenase from *Rhodococcus globerulus* PWD1 [31], and DbfB 2,2′,3-trihydroxybiphenyl dioxygenase from *Sphingomonas* sp. RW1 [30].

Finally, we demonstrate that (*2E*,*4E*)-2,4-dihydroxy-6-oxonona-2,4-dienedioic acid, the ring cleavage product of HcdB protein, is subsequently hydrolyzed by the putative HcdC hydroxymuconic semialdehyde hydrolase. This enzyme has a low sequence homology to any of the previously characterized enzymes from *Rhodococcus* sp. V49 [52], *E. coli* K-12 [53], *Comamonas testosteroni* TA441 [55], or *Rhodococcus globerulus* PWD1 [31], therefore further investigation is needed to elucidate the exact mechanism of the hydrolysis and the specificity of the HcdC enzyme for substrates.

In summary, here we report a 7-hydroxycoumarin catabolic pathway in *Pseudomonas* sp. 7HK4 bacteria. New metabolites and genes responsible for the degradation of 3-(2,4-dihydroxyphenyl)-propionic acid have been isolated and identified. Our results show that the degradation of 7-hydroxycoumarin in *Pseudomonas* sp. 7HK4 involves a distinct metabolic pathway, compared to the previously characterized coumarin catabolic routes in *Pseudomonas*, *Arthrobacter,* and *Aspergillus* species [9,10,11,12,13,14,15,16]. It has been shown that *Pseudomonas* sp. 7HK4 bacteria employ unique flavin-binding *ipso*-hydroxylase for the oxidation of the aromatic ring of 3-(2,4-dihydroxyphenyl)-propionic acid. None of the proteins described in this paper have substantial sequence homology to the previously characterized enzymes implicated in the degradation of structurally similar substrates, such as 3-(2-hydroxyphenyl)-propionic acid in *Rhodococcus* sp. V49 [52], 3-(3-hydroxyphenyl)-propionic acid and 3-hydroxycinnamic acid in *E. coli* K-12 [53], *Comamonas testosteroni* TA441 [54], *Rhodococcus globerulus* PWD1 [31], or even 4-hydroxyphenylacetate in *Escherichia coli* W [64]. Thus, our results provide a fundamentally new insight into the degradation of hydroxycoumarins by the soil microorganisms. In addition, the discovered new bacteria and enzymes can be further employed for the development of novel biocatalytic processes useful for industry.

## 4. Materials and Methods

### 4.1. Bacterial Strains, Plasmids, and Reagents

*Pseudomonas* sp. 7HK4 bacterial strain capable of using 7-hydroxycoumarin as the sole source of carbon and energy was isolated from soil by enrichment in mineral medium containing 0.05% of 7-hydroxycoumarin. For cloning purposes, *E. coli* DH5α bacteria (φ80 *lacZ*ΔM15 Δ(*lacZY-argF*)*U169 deoR recA1 endA1 hsdR17*(r_K_-m_K_+) *supE44 thi-1 gyrA96 relA1*) (Thermo Fischer Scientific, Lithuania) were used. *E. coli* BL21 (DE3) bacteria (F- *ompT gal dcm lon hsdS*_B_(r_B_-m_B_-) λ(DE3) [*lacI lacUV5-T7* gene 1, *ind1*, *sam7*, *nin5*]) (Novagen, Darmstadt, Germany) were used for gene expression studies.

All reagents used during this study are listed in Table S1 and plasmids are described in Table S2 in the Supplementary Material.

### 4.2. Bioconversions with Whole Cells

*Pseudomonas* sp. 7HK4 bacteria were grown in mineral medium supplemented with 0.05% (*w*/*v*) of 7-hydroxycoumarin or glucose, as the sole carbon and energy source, at 30 °C with rotary aeration (180 rpm) for 48 h. *E. coli* BL21 (DE3) bacteria containing recombinant genes were grown in 30 mL of Brain Heart Infusion (BHI) medium at 30 °C and 180 rpm overnight. High density bacterial culture was centrifuged and resuspended in 30 mL of minimal C-750501 medium, in which the synthesis of proteins was induced with 1 mM of isopropyl-*β*-D-1-thiogalactopyranoside (IPTG) after 1.5 h of incubation at 20 °C and 180 rpm [65]. Incubation at 20 °C was continued for another 24 h. Both *Pseudomonas* sp. 7HK4 and *E. coli* cells were sedimented by centrifugation (3220× *g*, 15 min). The collected cells were washed twice with 15 mL of 0.9% NaCl solution. For whole-cell conversion experiments, cells from 20 mL of culture were resuspended in 1 mL of 50 mM potassium phosphate buffer (pH 7.2). All small-scale bioconversions with whole cells were made in 50 mM potassium phosphate buffer, pH 7.2, which contained 1–2 mM of the substrate. The reaction mixtures were kept in a thermoblock at 30 °C and 500 rpm. Bioconversion mixtures were centrifuged for 2 min at 10,000× *g*, and 100 µL of the supernatant were analyzed by UV-VIS spectroscopy (range 200–600 nm). Measurements were repeated to record the changes in the absorption intensity over time. All measurements were performed with PowerWave XS microplate reader.

### 4.3. Preparation of cell-free extracts

Cells were sedimented by centrifugation (3220× *g*, 15 min). The biomass was resuspended in 3 mL of 50 mM potassium phosphate buffer (pH 7.2). The cells were disrupted by pulse-mode sonication (3 min duration and 1 s cycles) at 4 °C. Cell debris was removed by centrifugation (4 °C, 16,100× *g*, 15 min).

### 4.4. Protein Purification

Proteins were purified with Äkta purifier 900 chromatography systems (GE Healthcare, Helsinki, Finland). Cell-free extracts were loaded onto a Ni^2+^ Chelating HiTrapTM HP column (1–5 mL) (GE Healthcare, Finland) equilibrated with 50 mM potassium phosphate buffer, pH 7.2, at 1.0 mL/min. The column was washed with at least 3 volumes of the same buffer. Then the bound proteins were eluted with 0.5 M imidazole in 50 mM potassium phosphate buffer, pH 7.2. The fractions containing the purified enzyme were combined and dialyzed against the 50 mM potassium phosphate buffer, pH 7.2, at 4 °C overnight. Proteins were analyzed by SDS-PAGE according to Laemmli [66]. Protein concentration was determined by the Lowry method [67].

### 4.5. Preparation of Proteins from a Polyacrylamide Gel for Mass Spectrometric Analysis

Proteins were fractionated on a SDS-polyacrylamide gel. After Coomassie blue R-250 staining, protein samples were extracted from the gel as described in Reference [68] with minor changes. Protein bands were excised from the gel with a razor, and the gel was then destained twice with 200 µL of 25 mM ammonium bicarbonate and 50% acetonitrile solution for 30 min at 37 °C. Protein disulfide bonds were reduced with 40 µL of 10 mM dithiothreitol (DTT) for 45 min 60 °C, followed by incubation with 30 µL of 100 mM iodoacetamide for 1 h at room temperature in the dark to alkylate free cysteines. Gel slices were washed again twice with 100 µL 25 mM ammonium bicarbonate and 50% acetonitrile solution for 15 min at 37 °C, dehydrated by adding 50 µL 100% acetonitrile and dried using a vacuum centrifuge. Gel pieces were incubated with up to 40 µL of activated trypsin (10 ng/µL) at 37 °C overnight. The next day, the supernatant was saved and the peptides were extracted from the gel by incubating gel slices in two consecutive changes of 50 µL of 5% trifluoroacetic acid and 50% acetonitrile solution for 1 h at 37 °C. Combined supernatants were dried using a vacuum centrifuge at 30 °C. Lyophilized peptides were dissolved in 20 µL of 0.1% trifluoroacetic acid solution. Peptides purified and concentrated using Millipore C18 ZipTips.

### 4.6. Enzyme Assay

Activity of 3-(2,4-dihydroxyphenyl)-propionic acid hydroxylase was measured spectrophotometrically by monitoring absorption changes of the reaction mixture at 340 nm wavelength due to the oxidation of either NADH or NADPH (ε340 = 6220 M^−1^ cm^−1^) after the addition of the substrate. The activity measurements were made with cell-free extracts or the purified protein. All measurements of the enzyme activity were carried out at room temperature in 1 mL of reaction mixture, containing 25–50 mM tricine or potassium phosphate buffers (pH 7.8), 100 µM NADH or NADPH, and 150 µM aromatic substrate. One unit of activity was defined as the amount of the enzyme that catalyzed the oxidation of 1 µmol of NADH or NADPH per minute.

### 4.7. In vivo Bioconversion of 3-(2,4-Dihydroxyphenyl)-propionic Acid

*E. coli* BL21 (DE3) bacteria, containing p4pmPmo and pTHPPDO plasmids, were grown in 200 mL of BHI medium at 30 °C and 180 rpm overnight. High density bacterial culture was centrifuged and resuspended in 200 mL of minimal C-750501 medium, in which synthesis of proteins was induced with 1 mM of IPTG at 20 °C and 180 rpm [65]. After 24 h of induction 3-(2,4-dihydroxyphenyl)-propionic acid was added to the final concentration of 4 mM. Bioconversion mixture was incubated for another 3 days at 30 °C with shaking. Cells were removed by centrifugation for 30 min at 3220× *g* and the supernatants were kept at 4 °C.

### 4.8. Purification of 3-(2,4-Dihydroxyphenyl)-propionic Acid Bioconversion Product

The supernatant containing the 3-(2,4-dihydroxyphenyl)-propionic acid oxidation product was incubated with 1.2 M ammonium chloride at room temperature overnight [39,50]. Reaction mixture was concentrated to ~100 mL volume and adjusted to pH 1 with concentrated HCl. The remains of substrate were extracted by five consecutive changes of 25 mL of ethyl acetate, and then aqueous fraction was purified using a reverse phase C_18_ column, equilibrated with water. Column was washed with at least 100 mL of water and then eluted with linear gradient of 0–60% methanol solution at a flow rate of 2 mL/min. Aqueous fractions were combined and evaporated (40 °C). Brownish crystals were dissolved in 0.1% formic acid solution and again loaded onto a reverse phase C_18_ column, previously equilibrated with 0.1% formic acid solution. Column was washed with at least 30 ml of 0.1% formic acid solution and then eluted with 60% methanol solution. Picolinic acid derivative was eluted with 0.1% formic acid solution. Fractions containing the product were collected, combined, and evaporated (40 °C). Picolinic acid yield from 145 mg of 3-(2,4-dihydroxyphenyl)-propionic acid fermentation was 34 mg, 24% of the theoretical yield. The product had traces of formic acid impurities, which aided the dissolution of the analyte in D_2_O for NMR analysis.

### 4.9. High-Performance Liquid Chromatography and Mass Spectrometry

Before the analysis, the samples were mixed with an equal part of acetonitrile and centrifuged. High-performance liquid chromatography and mass spectrometry (HPLC-MS) was carried out using the system, consisting of the CBM-20 control unit, two LC-2020AD pumps, SIL-30AC auto sampler, and CTO-20AC column thermostat, using the SPD-M20A detector and LCMS-2020 mass spectrometer with ESI source (Shimadzu, Kyoto, Japan).

Chromatographic fractionation was conducted using YMC-Pack Pro C_18_ column, 150 × 3 mm (YMC, Kyoto, Japan) at 40 °C, with 0.1% formic acid solution in water and acetonitrile gradient from 5% to 95%.

Mass spectra were recorded from *m*/*z* 10 up to 500 *m*/*z* at 350 °C and ±4500 V using N_2_. Mass spectrometry analysis was carried out using both the positive and negative ionization modes. The data were analyzed using LabSolutions LC/MS software (Shimadzu, Japan).

### 4.10. Nuclear Magnetic Resonance Spectroscopy

In total, 20 mg of the sample was dissolved in 0.5 mL D_2_O. NMR spectra were recorded on an Ascend 400: ^1^H NMR – 400 MHz, ^13^C NMR – 100 MHz (Bruker, Billerica, MA, USA). Chemical shifts are reported in parts per million relative to the solvent resonance signal as an internal standard.

### 4.11. Protein MS-MS Analysis

Peptides were subjected to de novo sequencing based on matrix-assisted laser desorption ionization time of flight (MALDI-TOF/TOF) mass spectrometry (MS) and subsequent computational analysis at the Proteomics Centre of the Institute of Biochemistry, Vilnius University (Vilnius, Lithuania). The sample was purified as described previously in Materials and Methods. Tryptic digests (0.5 µL) were transferred on 384-well MALDI plate with 0.5 µL 4 mg/mL α-cyano-4-hydroxycinnamic acid (CHCA) matrix in 50% acetonitrile with 0.1% trifluoroacetic acid and analyzed with an Applied Biosystems/MDS SCIEX 4800 MALDI TOF/TOF™ mass spectrometer. Spectra were acquired in the positive reflector mode between 800 and 4000 *m*/*z* with fixed laser intensity at 3700 (Laser shots: 400; Mass accuracy: ±50 ppm). The most intense peaks of each survey scan (MS) were fragmented for sequence analysis (Collision energy: 1 keV; CID: no CID or medium air pressure CID used; Laser intensity: 4200–4400; Laser shots: 500–1000; Fragment mass accuracy: ±0.1 Da). Sequence analysis and peak lists were generated using GPS Explorer™ De Novo Explorer.

### 4.12. DNA Sequencing and Accession Numbers

The genome sequencing by Illumina (paired-ends) and assembling (in total, 35 contigs) was carried at Baseclear (Leiden, The Netherlands). The FASTQ sequence reads were generated using the Illumina Casava pipeline version 1.8.3. Initial quality assessment was based on data passing the Illumina Chastity filtering. Subsequently, reads containing adapters and/or PhiX control signal were removed using an in-house filtering protocol. The second quality assessment was based on the remaining reads using the FASTQC quality control tool version 0.10.0. The number of reads was 5,605,132 and an average quality score (Phred) was 37.72.

The accession number for partial 16S ribosomal RNA nucleotide sequence of *Pseudomonas* sp. 7HK4 is MH346031. Accession numbers for the sequences of *hcdA*, *hcdB,* and *hcdC* genes are MH346032, MH346033, MH346034, respectively.

## Supplementary Materials

The following are available online, Table S1: Materials and reagents used in the studies, Table S2: Plasmids used in the studies, Table S3: The list of primers used in this study, Figure S1: Phylogenetic tree of *Pseudomonas* sp. 7HK4 bacteria based on partial 16S rDNA sequences, Figure S2: Biotransformation of 7-hydroxycoumarin, 6-hydroxycoumarin, coumarin and 6,7-dihydroxycoumarin by whole cells of *Pseudomonas* sp. 7HK4, Figure S3: Phylogenetic trees of HcdA, HcdB and HcdC proteins, Figure S4: SDS-PAGE, UV/Vis spectrum and analytical gel filtration chromatography of purified HcdA protein, Figure S5: Specificity of HcdA protein to flavin and nicotinamide cofactors, Figure S6: Activity of HcdA protein in different buffer systems, Figure S7: Activity of HcdA protein in different pH, Figure S8: Kinetic analysis of HcdA as determined by NADH oxidation for 3-(2,4-dihydroxyphenyl)-propionic acid, Figure S9: Kinetic analysis of HcdA as determined by NADH oxidation for NADH, Figure S10: Double reciprocal plot of NADH oxidation as a function of NADH concentration, Figure S11: Biotransformations of 3,4-dihydroxybenzoic acid, 2′,3′-dihydroxy-4′-methoxyaceto-phenone hydrate, gallacetophenone, pyrogallol, 2,3,4-trihydroxybenzoic acid and 2,3,4-trihydroxy-benzophenone by whole cells of *E. coli* BL21 containing *hcdB* gene, Figure S12: ^1^H NMR spectrum of 6-(2-carboxyethyl)-4-oxo-1,4-dihydropyridine-2-carboxylic acid, Figure S13: ^13^C NMR spectrum of 6-(2-carboxyethyl)-4-oxo-1,4-dihydropyridine-2-carboxylic acid, Figure S14: Resonance structure of *oxo*-picolinic acid derivative showing electron densities on aromatic carbons, Figure S15: SDS-PAGE of *E. coli* BL21 cell-free extract, containing induced recombinant HcdA, HcdB and HcdC proteins, Figure S16: HPLC-MS analysis of 3-(2-hydroxyphenyl)-propionic acid bioconversion mixture.
