# Supplementary material for: Biodegradation of 7-Hydroxycoumarin in Pseudomonas mandelii 7HK4 via ipso-Hydroxylation of 3-(2,4-Dihydroxyphenyl)-propionic Acid
Source: Molecules. 2018 Oct 12;23(10):2613. doi: 10.3390/molecules23102613 (PMC6222606; doi:10.3390/molecules23102613)
Supplement: Supplementary file 1 [file molecules-23-02613-s001.pdf]

Biodegradation of 7-hydroxycoumarin in *Pseudomonas mandelii* 7HK4 via *ipso*-hydroxylation of 3-(2,4-dihydroxyphenyl)-propionic acid

Arūnas Krikštaponis, Rolandas Meškys

Department of Molecular Microbiology and Biotechnology, Institute of Biochemistry, Life Sciences Center, Vilnius University, Sauletekio al. 7, Vilnius LT-10257, Lithuania

Supplementary Information

Table S1. Materials and reagents used in the studies.

Chemicals and reagents	Source
7-Hydroxycoumarin, ethyl acetate, methanol	Merk
Ampicillin, streptomycin, 3-(2-hydroxyphenyl)-2-propenoic acid, 3-(4-hydroxyphenyl)-2-propenoic acid, 3-(3-hydroxyphenyl)-2-propenoic acid, pyrocatechol, coumarin, cinnamyl alcohol, 3-(2,4-dihydroxyphenyl)-propionic acid, 2-ethylphenol, caffeic acid	Fluka
3-Hydroxycoumarin, 4-hydroxycoumarin, 7-methylcoumarin, 3-methylcatechol, 4-methylcatechol, iodoacetamide, trifluoroacetic acid, kanamycin sulfate, 3-(2-hydroxyphenyl)-propionic acid, *trans*-2,4-dihydroxycinnamic acid, 3-(2-bromophenyl)-propionic acid, 3-(2-nitrophenyl)-propionic acid, 3-phenylpropionic acid, *trans*-cinnamic acid, 2-propylphenol, 2-propenylphenol, *o*-cresol, *o*-tyrosine, resorcinol, 2,3-dihydroxypyridine, 2-hydroxy-4-aminopyridine, *N*-methyl-2-pyridone, *N*-ethyl-2-pyridone, *N*-propyl-2-pyridone, *N*-butyl-2-pyridone, indoline, indole, pyrogallol, 3-methoxycatechol, 2‘,3‘-dihydroxy-4‘-methoxyacetophenone hydrate, gallacetophenone, 3,4-dihydroxybenzoic acid, 2,3,4-trihydroxybenzoic acid, 2,3,4-trihydroxybenzophenone, 1,2,4-benzentriol, 6,7-dihydroxycoumarin	Sigma-Aldrich
E-2,4-dihydroxycinnamic acid, 3-(2,3-dihydroxyphenyl)-propionic acid	This study
Succinic acid, glucose	Labochema
Agar, Brain Heart Infusion Broth (Bhi), Lysogeny broth (LB)	Oxoid

Restriction endonucleases, Phusion High-Fidelity PCR Master Mix with HF Buffer, Isopropyl β-D-1-thiogalactopyranoside (IPTG), PageRuler Prestained Protein Ladder	Thermo Fischer Scientific Baltics
RapidClean Resin	Advansta
C_{18} Reverse-Phase column (12 g)	Grace

Table S2. Plasmids used in the studies.

**Plasmids**	**Properties**	**Source**
pET21b(+)	Amp^R, *lacI*, $P_{T7\textit{lac}}$, 5442 bp	Novagen, Germany
pET28b(+)	Kan^R, *lacI*, $P_{T7\textit{lac}}$, 5368 bp	Novagen, Germany
pCDFDuet-1	Sm^R, *lacI*, $P_{T7\textit{lac}}$, 3781 bp	Novagen, Germany
pTHPPDO	The *hcdB* gene is cloned into pET28b(+), *Nco*I and *Hind*III restriction sites	This study
p4pmPmo	The *hcdA* gene is cloned into pET21b(+), *Nde*I and *Xho*I restriction sites	This study
p4pmPmoH^c	The *hcdA* gene is cloned into pET21b(+), with C-terminal His$_6$-tag, *Nde*I and *Hind*III restriction sites	This study
p2K4PH	The *hcdC* gene is cloned into pET21b(+), *Nde*I and *Xho*I restriction sites	This study

pCDF-BC	The *hcdB* and *hcdC* genes are cloned into pCDFDuet-1, *Nco*I and *Hind*III, or *Nde*I and *Xho*I restriction sites, respectively	This study
p5Pmo	The gene of 3-(2-hydroxyphenyl)-propionic acid monooxygenase from *Rhodococcus* sp. K5 is cloned into pET21b(+)	This study

Table S3. The list of primers used in this study.

**Primers**	**Primer sequence, 5'-3'**	**Features, target**	**Source**
hcdA_F	gtaattccatatggactacgatgtcatcat	*Nde*I restriction site, *hcdA* gene	This study
hcdA_R1	aaaccaagcttctggcttagtccctg	*Hind*III restriction site, *hcdA* gene	This study
hcdA_R2	aaaattctcgagttactggcttagtccctg	*Xho*I restriction site, STOP codon, *hcdA* gene	This study
hcdB_F	catgccatgggtatgcccgcattaccgactat	*Nco*I restriction site, *hcdB* gene	This study
hcdB_R	aacccaagcttcagccgattcgaacccg	*Hind*III restriction site, STOP codon, *hcdB* gene	This study
hcdC_F	gtaattccatatgaagcttatttcgtaccg	*Nde*I restriction site, *hcdC* gene	This study

hcdC_R	aaaattctcgagttaggcttcgtcaataacgc	*Xho*I restriction site, STOP codon, *hcdC* gene	This study
Woo1	agagtttgatcmtggctc	16S rRNR gene	[1]
Woo2	gntaccttgttacgactt	16S rRNR gene	[1]

Multiplication of genes was conducted using Phusion High-Fidelity PCR Master Mix with HF Buffer, following the user manuals provided by manufacturer of reagents.

Amplification conditions:

(a) *hcdA* gene: initial denaturation for 1 min at 98°C, then 40 cycles of denaturation for 10 s at 98°C, annealing for 20 s at 69°C, and extention for 50 s at 72°C, final extension for 5 min at 72°C;

(b) *hcdB* and *hcdC* genes: initial denaturation for 1 min at 98°C, then 40 cycles of denaturation for 10 s at 98°C, annealing for 15 s at 60°C, and extention for 30 s at 72°C, final extension for 5 min at 72°C

Bacterial culture media

Mineral medium (pH 7.2): 5 g/L NaCl, 1 g/L $NH_4H_2PO_4$, 1 g/L K_2HPO_4, 0,4 g/L $MgSO_4 \cdot 7H_2O$.

Minimal C-750501 medium (pH 8.0) [2].

LB medium (pH 7.2): 20 g of powder in 1 L of water.

BHI medium (pH 7.4): 37 g of powder in 1 L of water.

For the production of agar plates 15 g of agar powder was added to 1 L of medium.

All media were sterilized for 30 minutes at 121°C, 1 atm.

Biochemical characterization of bacteria

Bacteria were characterized by using API strips according user manuals (Biomerieux, USA). API 50 CH strip was used for carbohydrate fermentation test. *Pseudomonas mandelii* 7HK4 bacteria were grown overnight in 10 mL of LB medium. Cells were centrifuged for 10 minutes at 3,220 × *g* and resuspended in 10 mL of API 50 CHB/E medium. 100 µL of resuspended culture was transferred into wells of API 50 CH strip and incubated at 30°C for 48 h. Color of the medium changes from red color to yellow color due to acid production, if the test is positive.

And API 20 ZYM strip was used to test enzyme activities. 10 mL of overnight bacterial culture was resuspended in 3 mL of mineral medium (without Mg^{2+}) and aliquoted into wells of API 20 ZYM strip. Incubated at 30°C for 4 h. After incubation 1 drop of ZYM A and 1 drop of ZYM B reagents were added to each well. Colorless wells show negative test results.

Biochemical analysis by API 50CH:

Acid is not produced from glycerol, erythritol, D-arabinose, L-arabinose, ribose, D-xylose, L-xylose, adonitol, methyl-xyloside, mannitol, galactose, D-glucose, D-fructose, D-mannose, L-sorbose, dulcitol, rhamnose, inositol, sorbitol, α-methyl-D-mannoside, α-methyl-D-glucoside, N-acetyl-glucosamine, amygdalin, arbutin, esculin, salicin, cellobiose, maltose, lactose, melibiose, saccharose, trehalose, inulin, melezitose, D-raffinose, amidon, glycogen, xylitol, gentiobiose, D-turanose, D-lyxose, D-tagatose, D-fucose, L-fucose, L-arabitol, D-arabitol, gluconate, 2-keto-gluconate, 5-keto-gluconate.

Biochemical analysis by API 20 ZYM:

Activities for esterase lipase (C8), β-galactosidase, β-glucosidase, esterase (C4), α-galactosidase, lipase (C4), cystine arylamidase, α-chymotrypsin, β-glucuronidase, α-mannosidase, α-fucosidase, alkaline phosphatase, leucinearylamidase, valnearylamidase, trypsin, acid phosphatase, α-glucosidase, N-acetyl-β-glucosaminidase are absent. Activity for naphthol-AS-BI-phosphohydrolase is present.

Synthesis of *E*-2,4-dihydroxycinnamic acid and 3-(2,3-dihydroxyphenyl)-propionic acid

The starting material 7-hydroxycoumarin (3.24 g, 20 mmol) was dissolved in a 2 M KOH solution (50 mL) and stirred for 2 h at 80–90°C temperature. Completion of the reaction was determined by thin layer chromatography (TLC, chloroform/methanol, 9/1). After the reaction was completed (TLC), the reaction mixture was diluted with water (100 mL) and then acidified to pH 3–4 with HCl. The acidic compounds were extracted with ethyl acetate. The organic solvent was dried (Na_2SO_4) and removed under reduced pressure. The residue was purified by column chromatography (silica gel, chloroform/methanol mixture). The solvents were removed under reduced pressure to afford 1.98 g (11 mmol, 55 % yield) of *E*-2,4-dihydroxycinnamic acid. MS (ESI^+): *m/z* 181.00 $[M+H]^+$; 179.00 $[M-H]^-$. ^{1}H NMR (DMSO-d_6, 400 MHz): δ = 3.37 (bs, 2H, OH-2, OH-4), 6.26 (dd, *J* = 8.5, 2.3 Hz, 2H, H-6), 6.28 (d, *J* = 16.0 Hz, 1H, H-7), 6.36 (d, *J* = 2.3 Hz, 1H, H-3), 7.38 (d, *J* = 8.6 Hz, 1H, , H-5), 7.71 (d, *J* = 16.1 Hz, 1H, H-8). ^{13}C NMR (DMSO-d_6, 100 MHz): δ = 102.95 (C-6), 108.17 (C-7), 113.19 (C-1), 114.64 (C-5), 130.54 (C-3), 140.38 (C-8), 158.74 (C-4), 161.13 (C-2), 169.06 (C-9).

3-(2,3-Dihydroxyphenyl)-propionic acid was converted from 3-(2-hydroxyphenyl)-propionic acid in *E. coli* BL21 whole cells. *E. coli* BL21 (DE3) bacteria, containing p5Pmo plasmid, were grown in 200 mL of BHI medium at 30 °C and 180 rpm overnight. High-density bacterial culture was centrifuged and resuspended in 200 mL of minimal C-750501 medium, in which synthesis of protein was induced with 1 mM of IPTG at 20 °C and 180 rpm. Incubation at 20 °C was continued for another 24 h. *E. coli* cells were sedimented by centrifugation (3,220 × *g*, 15 min). The collected cells were washed twice with 30 mL of 0.9% NaCl solution. Cells were resuspended in 100 mL of 50 mM potassium phosphate buffer (pH 7.2) containing 2 mM of 3-(2-hydroxyphenyl)-propionic acid and incubated for 48 h. Bioconversion product was analyzed by HPLC-MS. Analysis of the reaction product confirmed the formation of 3-(2,3-dihydroxyphenyl)-propionic acid, found $[M-H]^{-}$ mass was 181, and substrate was almost depleted (Figure S16). 3-(2,3-Dihydroxyphenyl)-propionic acid was not purified, and the whole cell-free bioconversion mixture was used further in conversions by *E. coli* BL21 (DE3) bacteria containing *hcdB* gene.

Gel filtration chromatography

The structure of the native HcdA protein was determined by gel filtration chromatography. The purified protein was applied to Superdex™ 200 10/300 GL column (GE Healthcare, Finland) using a 50 mM Tris-HCl buffer, pH 7.5, containing 0.1 M of NaCl at 0.3 mL/min. Protein molecular mass was determined using the calibration curve, constructed by the application of carbonic anhydrase (M=29 kDa), albumin (M=66 kDa) and apoferritin (M=443 kDa). 0.8–1 mg of all proteins was dissolved in 0.5 mL 50 mM Tris-HCl, pH 7.5 and 0.1 M NaCl buffer. The K_{av} values were calculated for 3 proteins

using the equation $K_{av} = (V_e-V_0)/(V_c-V_0)$, where V_0 = column void volume = 8.2 mL, V_c = geometric column volume = 23.6 mL and V_e = elution volume for each protein: carbonic anhydrase (29 kDa) V_e=16.9 mL, albumin (66 kDa) V_e=14.47 mL and apoferritin (443 kDa) V_e=10.6 mL. For the sample, the observed V_e was used to calculate the corresponding K_{av} value that was used to determine the molecular weight from the equation of the calibration curve.

Kinetic characterization of HcdA hydroxylase

The specificity for both flavin and nicotinamide cofactors was investigated (Figure S5). The HcdA hydroxylase was able to utilize either NADH or NADPH, although the oxidation rates of NADPH were almost two-fold lower. The addition of FAD or FMN to the reaction mixtures showed no significant changes in NADPH oxidation, however additional FAD and FMN increased the oxidation rates of NADH by 6 to 12 %, respectively. The optimum reaction conditions for the HcdA activity was found to be a low ionic strength tricine buffer, pH 7.8–8.0 and 18–25ºC temperature (Figures S6–S7). The NADH oxidation assay was used to determine the kinetic parameters of HcdA. The K_m value for NADH calculated from the initial velocity analysis was 50.10 ± 3.50 µM in the presence of 500 µM 3-(2,4-dihydroxyphenyl)-propionic acid (Figure S8), and the apparent K_m for 3-(2,4-dihydroxyphenyl)-propionic acid was 13.00 ± 1.20 µM in the presence of 300 µM NADH (Figure S9), with k_{cat} of 7.91 ± 0.17 s^{-1}. Besides, the initial velocities were measured for HcdA with an excess of FMN varying both NADH and 3-(2,4-dihydroxyphenyl)-propionic acid concentrations using steady state kinetics and the NADH oxidation assay. The derived velocities were plotted using the double reciprocal

plots, which indicated the formation of a ternary complex since lines were not parallel but intersected in the upper left quadrant (Figure S10).

Purification of genomic DNA

Pseudomonas mandelii 7HK4 bacteria were grown overnight in 20 mL of LB medium containing 1 % of glycerol. Cells were centrifuged for 10 minutes at 3,220 x *g* and washed with 3 ml of 50 mM citrate buffer (pH 8.2). Cells were divided into 6 parts, each of them was resuspended in 600 µL of lysis buffer (50 mM Tris-HCl (pH 8.0), 50 mM EDTA, 3 % SDS, 1 % mercaptoethanol, 0.2 M NaCl) [3], also 15 µL of 20 mg/mL Proteinase K was added, and incubated for 2 hours at 65°C. Then lisates were centrifuged for 15 min at 16,100 × *g* and 300 µL of 7.5 M ammonium acetate (pH 6.0) was added to the supernatant, followed by mixing by inversion several times and centrifugation for 20 min at 16,100 × *g*. DNA was precipitated with 2 volumes of ethanol overnight at -20°C, followed by centrifugation. DNA precipitates were resuspended and combined in 60 µL of 20 mM Tris-HCl buffer (pH 8.0) and incubated with 10 µg RNase A. Genomic DNA was purified using Rapid Clean protein removal resin.

Analysis of DNA and protein sequences

DNA and protein sequences were analyzed using VectorNTI Advance 9.0 [4] and MEGA 5.0 [5,6], respectively. The search of homologues was conducted against NCBI database using BLAST [7]. Phylogenetic trees were constructed by MEGA version 5.0 application tool [5,6], using the Neighbor-joining method (N-J) [8] in accordance with the Maximum Composite Likelihood model for nucleotides or Poisson model for amino acids [9].

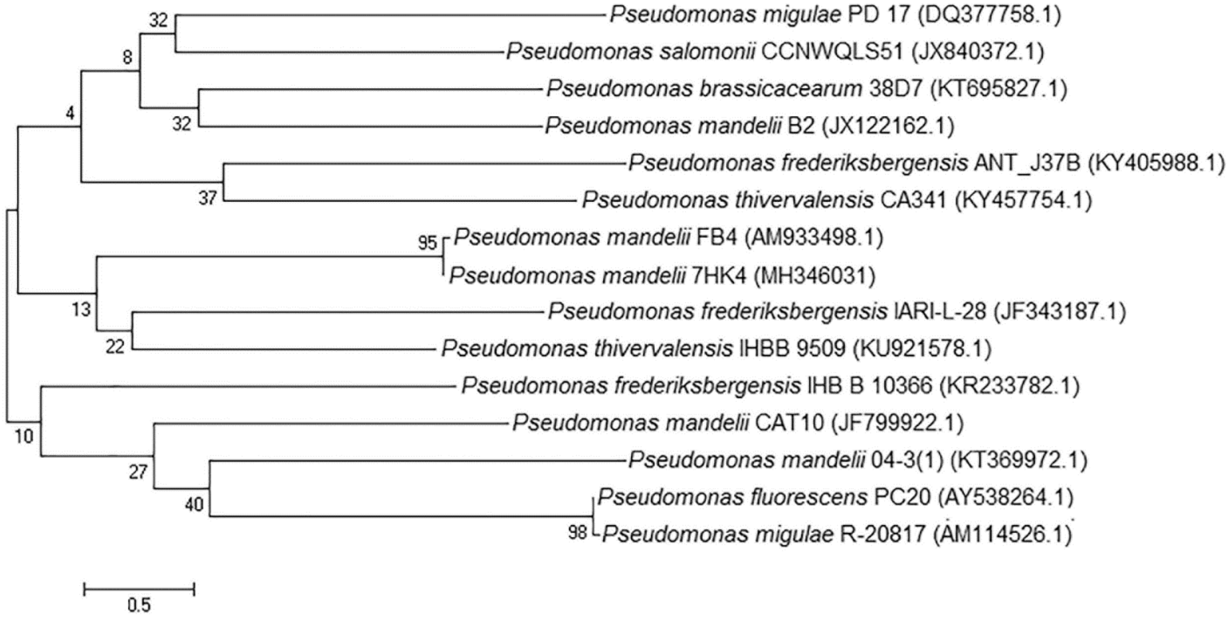

Figure S1. Phylogenetic tree of *Pseudomonas mandelii* 7HK4 bacteria based on partial 16S rDNA sequences. The numbers on the nodes indicate how often (no. of times, %) the species to the right grouped together in 1000 bootstrap samples. Bars represent the number of base substitutions per site. Accession numbers are given in parentheses.

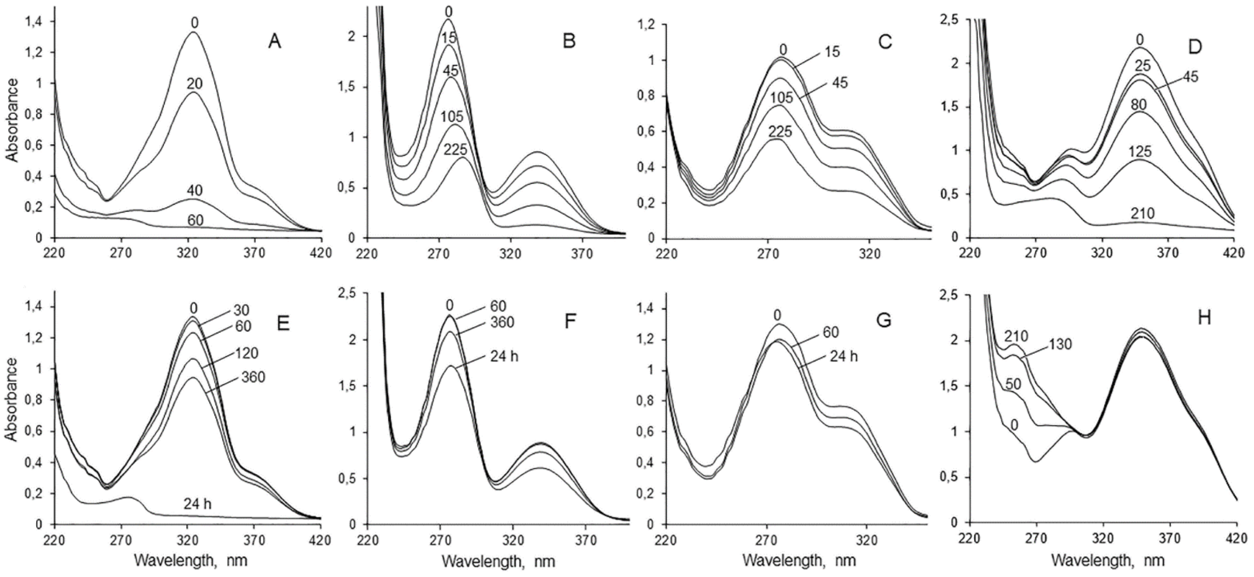

Figure S2. Biotransformation of 7-hydroxycoumarin (A–E), 6-hydroxycoumarin (B–F), coumarin (C–G) and 6,7-dihydroxycoumarin (D–H) by whole cells of *Pseudomonas mandelii* 7HK4. Cells were pre-grown with 7-hydroxycoumarin (A–D) and glucose (E–H). Biotransformations were carried out with bacterial culture (OD_{600} ~2) in 50 mM potassium phosphate buffer (pH 7.2) at 30°C with 0.5 mM of substrate. Incubation time is shown in min. 24 h – incubation for 24 hours.

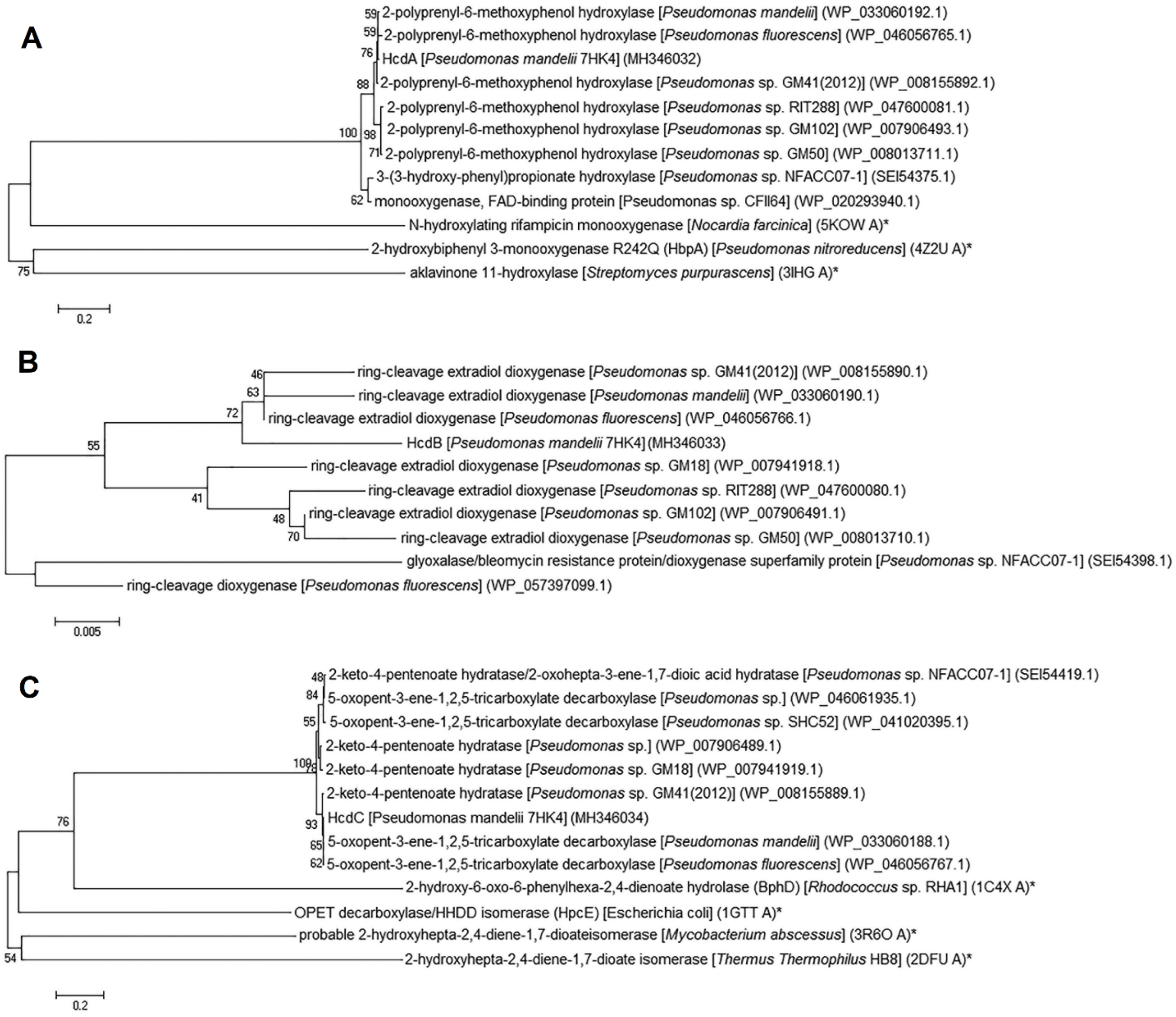

Figure S3. A. Phylogenetic tree of HcdA protein. Neighbor joining analysis was performed on the 8 closest homologues of HcdA and 3 other homologous proteins with

known structure and/or function. The numbers on the nodes indicate how often (no. of times, %) the species to the right grouped together in 1000 bootstrap samples. Bars represent the number of amino acid substitutions per site. Accession numbers are given in parentheses. Proteins with known structure and/or function are marked with an asterix (*). B. Phylogenetic tree of HcdB protein. Neighbor joining analysis was performed on the 9 closest homologues of HcdB. The numbers on the nodes indicate how often (no. of times, %) the species to the right grouped together in 1000 bootstrap samples. Bars represent the number of amino acid substitutions per site. Accession numbers are given in parentheses. C. Phylogenetic tree of HcdC protein. Neighbor joining analysis was performed on the 8 closest homologues of HcdC and 4 other homologous proteins with known structure and/or function. The numbers on the nodes indicate how often (no. of times, %) the species to the right grouped together in 1000 bootstrap samples. Bars represent the number of amino acid substitutions per site. Accession numbers are given in parentheses. Proteins with known structure and/or function are marked with an asterix (*).

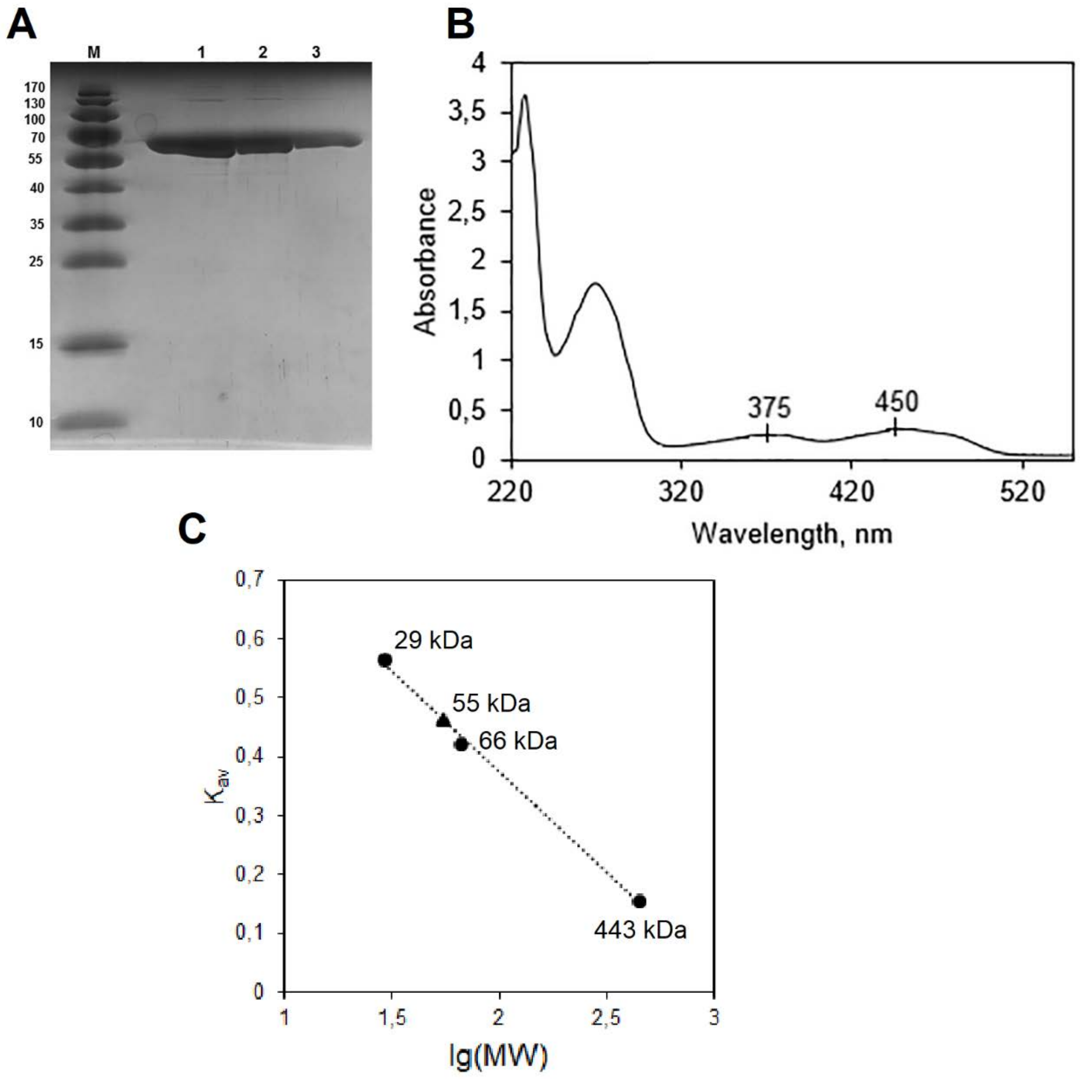

Figure S4. A. SDS-PAGE of His_6-tagged HcdA protein purified by affinity chromatography. Lane 1 – 1 µl of eluted protein, lane 2 – 0.5 µl of eluted protein, lane 3 – 0.25 µl of eluted protein. M – molecular mass ladder (kDa). B. UV/Vis spectrum of HcdA protein purified by affinity chromatography. C. Analytical gel filtration chromatography of HcdA protein. The calibration curve used to estimate the native molecular weight based on the elution position during analytical gel filtration is indicated. Filled circles – carbonic anhydrase (MW=29 kDa), albumin (MW=66 kDa) and apoferritin (MW=443 kDa); filled triangle – native HcdA protein.

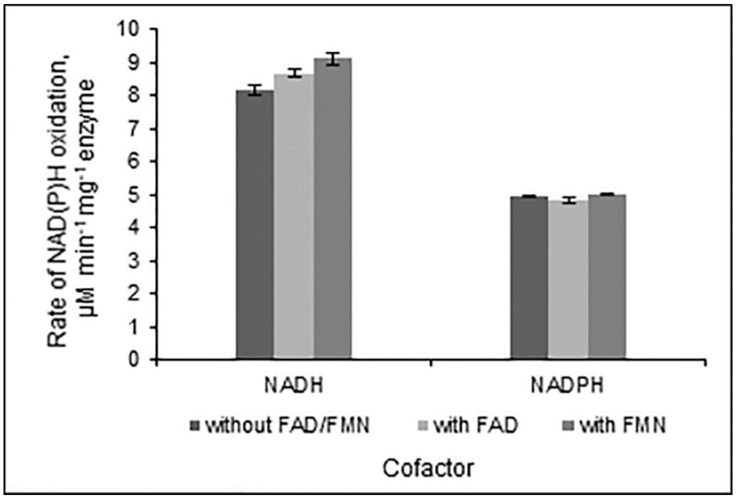

Figure S5. Specificity of HcdA protein to flavin and nicotinamide cofactors. Enzymatic assays were carried out in 50 mM tricine buffer (pH 7.8) with 40 nM HcdA enzyme and 50 µM of 3-(2,4-dihydroxyphenyl)-propionic acid, in presence of 75 µM NAD(P)H with/without 30 µM FAD/FMN at room temperature. Rates of NAD(P)H oxidation were observed at 340 nm wavelength. Experiment was performed in triplicate and error bars indicate standard error.

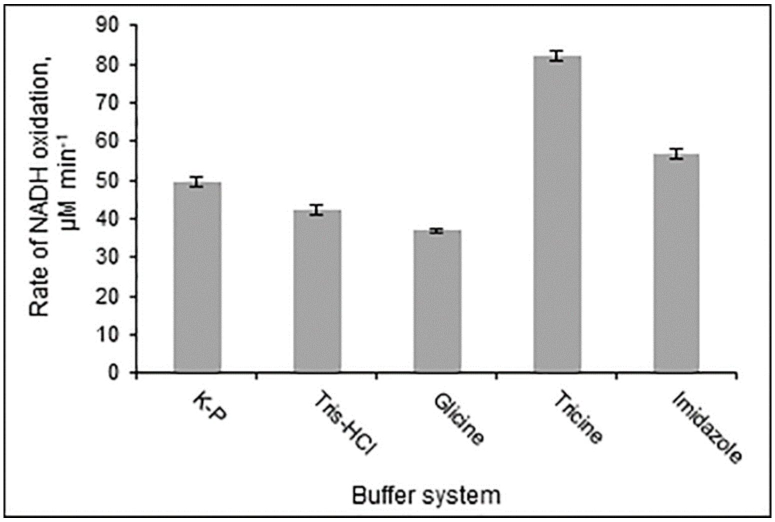

Figure S6. Activity of HcdA protein in different buffer systems. Enzymatic assays were carried out in 50 mM potassium phosphate (K-P), tris-HCl, glycine, tricine or imidazole buffers (pH 8.0) with excess of HcdA enzyme and 150 µM of 3-(2,4-dihydroxyphenyl)-

propionic acid, in presence of 100 µM NADH at room temperature. Rates of NADH oxidation were observed at 340 nm wavelength. Experiment was performed in triplicate and error bars indicate standard error.

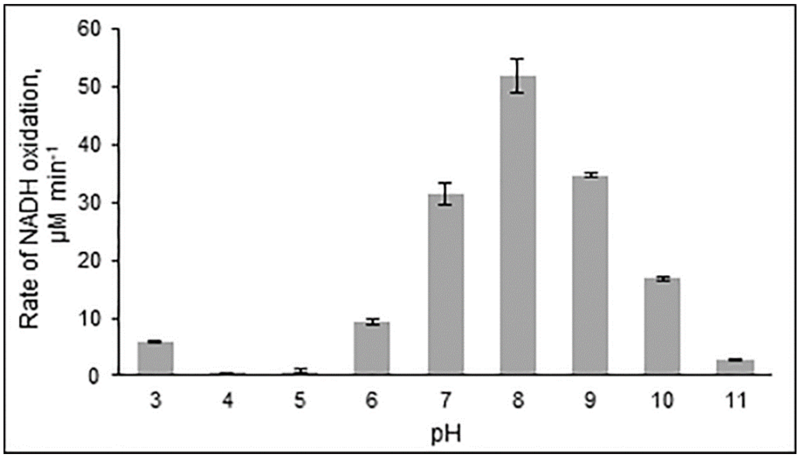

Figure S7. Activity of HcdA protein in different pH. Enzymatic assays were carried out in 50 mM potassium phosphate (K-P) buffer with excess of HcdA enzyme and 150 µM of 3-(2,4-dihydroxyphenyl)-propionic acid, in presence of 100 µM NADH at room temperature. Rates of NADH oxidation were observed at 340 nm wavelength. Experiment was performed in triplicate and error bars indicate standard error.

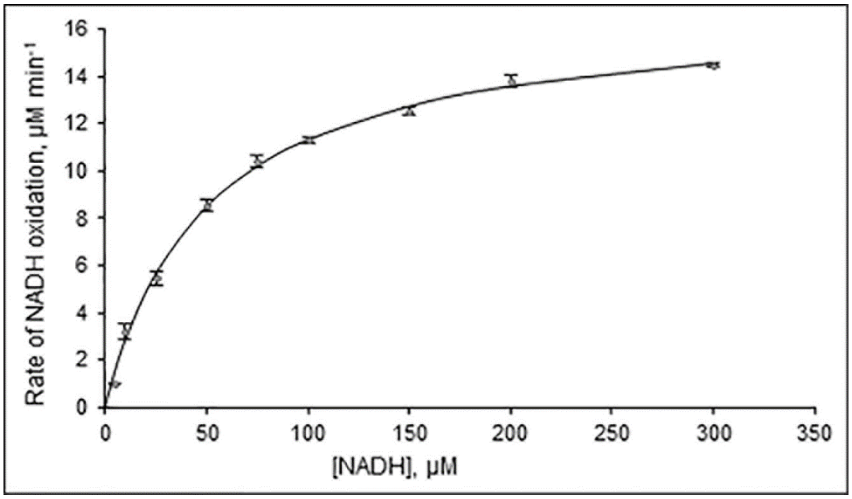

Figure S8. Kinetic analysis of HcdA as determined by NADH oxidation. Initial velocities were measured in the presence of 25 mM tricine buffer (pH 7.8) with 35.8 nM HcdA enzyme, 500 µM of 3-(2,4-dihydroxyphenyl)-propionic acid, 30 µM FMN and 5–300 µM NADH at room temperature. The curve for the NADH oxidation assay was fit to the standard equation for Michaelis-Menten reactions. Rates of NADH oxidation were observed at 340 nm wavelength. Experiment was performed in triplicate and error bars indicate standard error.

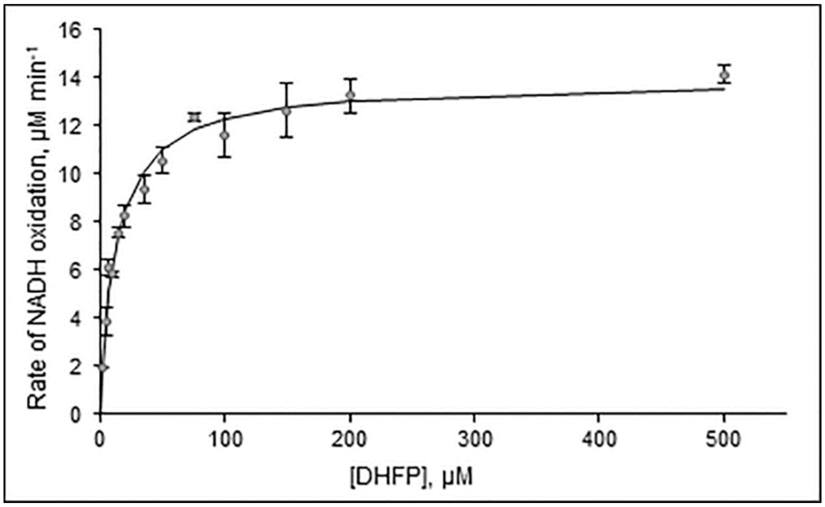

Figure S9. Kinetic analysis of HcdA as determined by NADH oxidation. Initial velocities were measured in the presence of 25 mM tricine buffer (pH 7.8) with 35.8 nM HcdA enzyme, 300 µM of NADH, 30 µM FMN and 2–500 µM 3-(2,4-dihydroxyphenyl)-propionic acid (DHFP) at room temperature. The curve for the NADH oxidation assay was fit to the standard equation for Michaelis-Menten reactions. Rates of NADH oxidation were observed at 340 nm wavelength. Experiment was performed in triplicate and error bars indicate standard error.

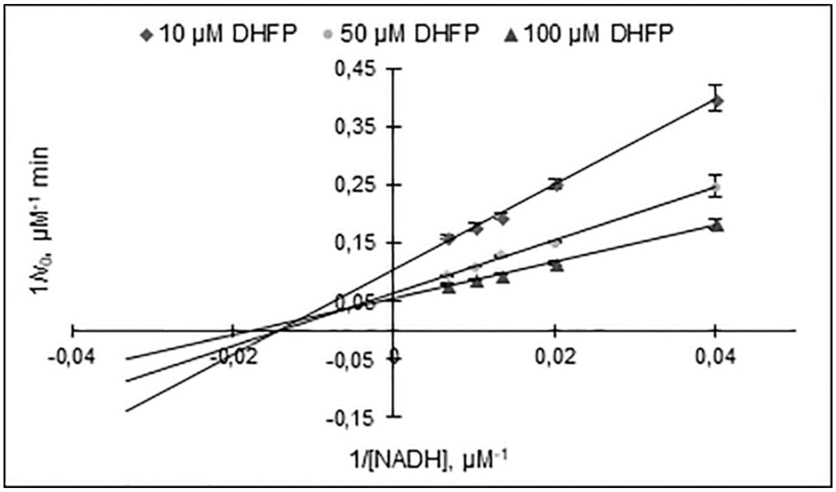

Figure S10. Double reciprocal plot of NADH oxidation as a function of NADH concentration. Ternary complex formation of FMN loaded HcdA with NADH and 3-(2,4-dihydroxyphenyl)-propionic acid. 3-(2,4-Dihydroxyphenyl)-propionic acid concentrations used were 10 µM (filled diamonds), 50 µM (filled circles), and 100 µM (filled triangles). Rates of NADH oxidation were observed at 340 nm wavelength. Experiment was performed in triplicate and error bars indicate standard error.

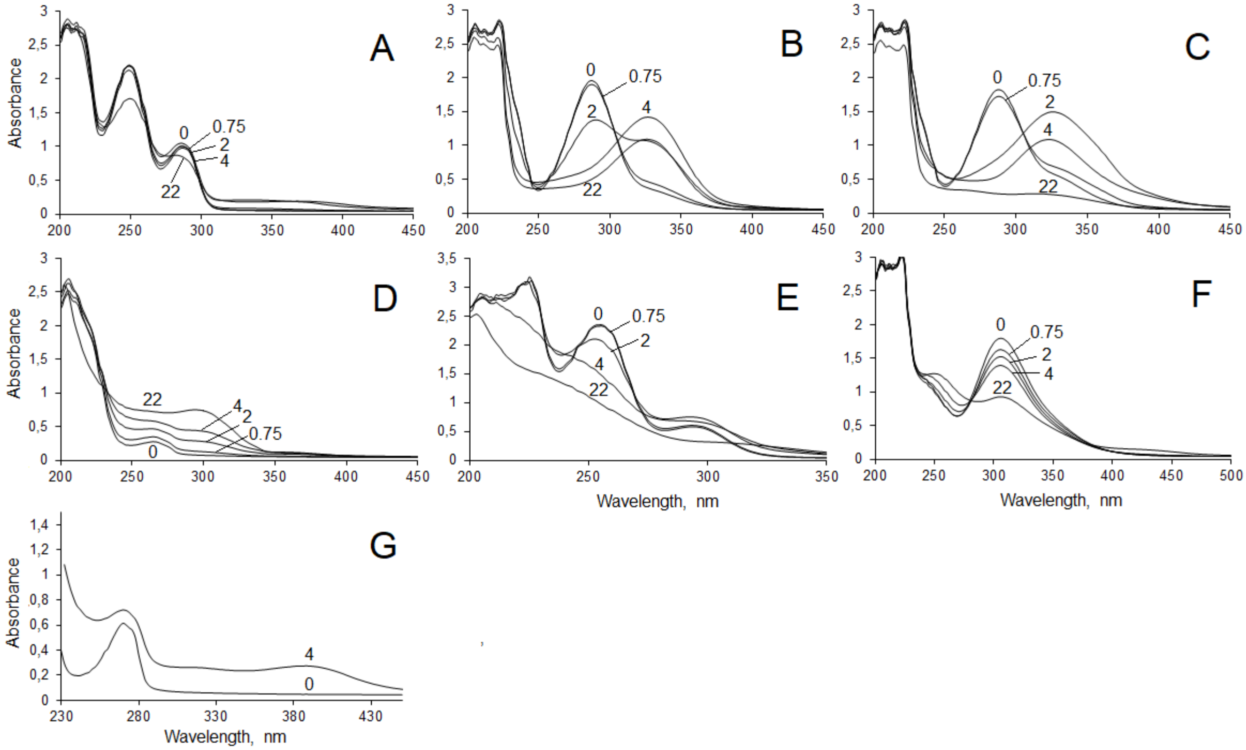

Figure S11. Biotransformations of 3,4-dihydroxybenzoic acid (A), 2',3'-dihydroxy-4'-methoxyaceto-phenone hydrate (B), gallacetophenone (C), pyrogallol (D), 2,3,4-trihydroxybenzoic acid (E), 2,3,4-trihydroxy-benzophenone (F) and 3-(2,3-dihydroxyphenyl)-propionic acid (G) by whole cells of *E. coli* BL21 containing *hcdB* gene. Biotransformations were carried out in 50 mM potassium phosphate buffer pH 7.5 at 30 °C with 0.5–2 mM of substrate. Incubation time is shown in hours.

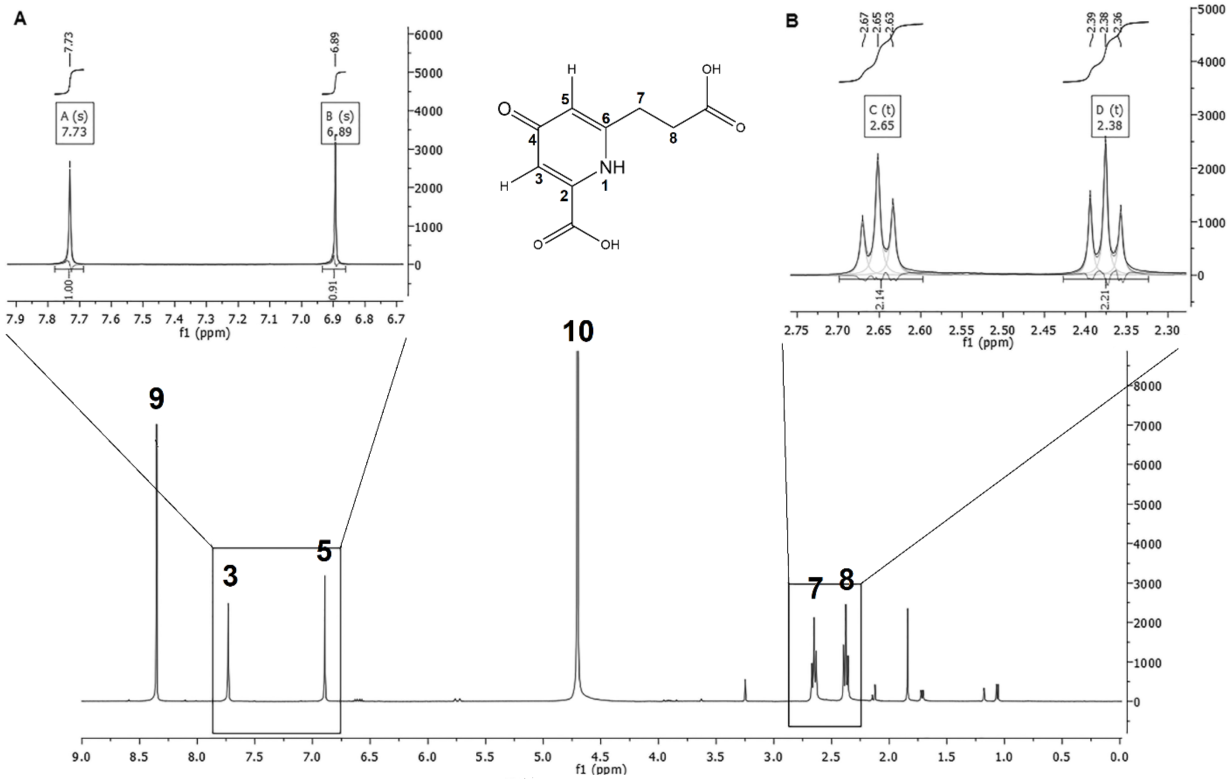

Figure S12. ^{1}H NMR spectrum (400 MHz, Deuterium Oxide) of 6-(2-carboxyethyl)-4-oxo-1,4-dihydropyridine-2-carboxylic acid. Identification of aryl (A) and methylene (B) protons. 9 – trace impurities of formic acid; 10 – solvent residual peak [10].

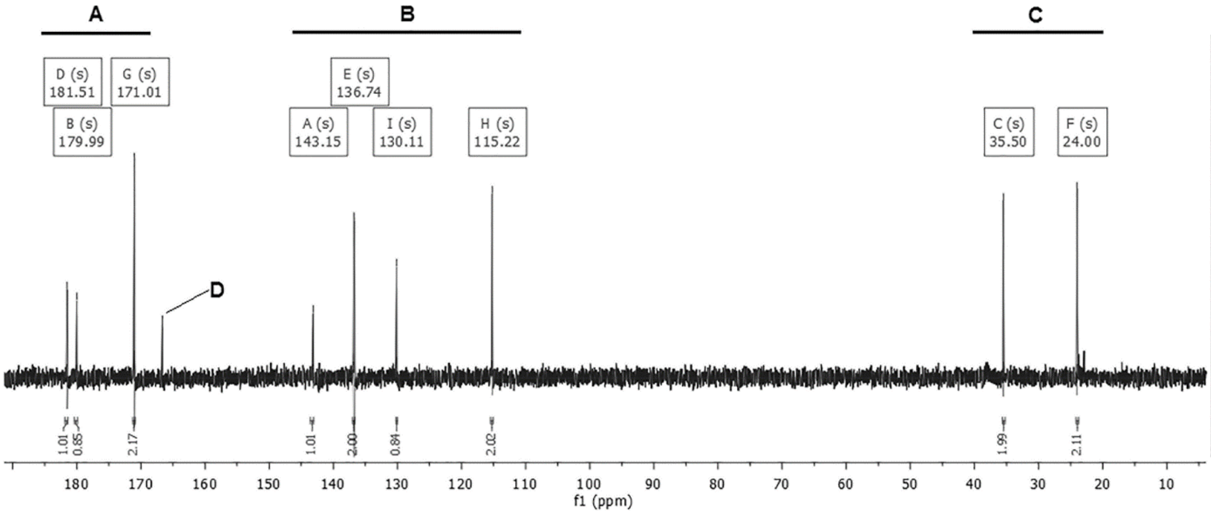

Figure S13. ^{13}C NMR spectrum (101 MHz, Deuterium oxide) of 6-(2-carboxyethyl)-4-oxo-1,4-dihydropyridine-2-carboxylic acid. Identification of carbonyl (A), aryl (B) and methylene (C) carbons. D – trace impurities of formic acid [10].

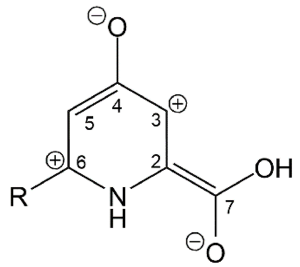

Figure S14. Resonance structure of oxo-picolinic acid derivative showing electron densities on aromatic carbons.

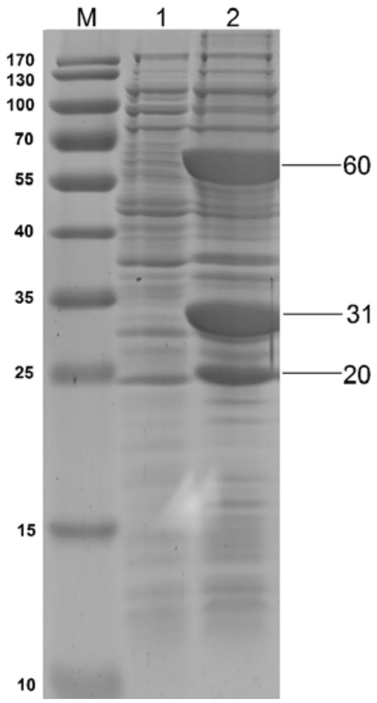

Figure S15. SDS-PAGE of *E. coli* BL21 cell-free extract, containing induced recombinant HcdA, HcdB and HcdC proteins (lane 2) and control cells without *hcdABC* genes (lane 1). M – molecular mass ladder (kDA). The arrows indicate HcdA, HcdB and HcdC proteins.

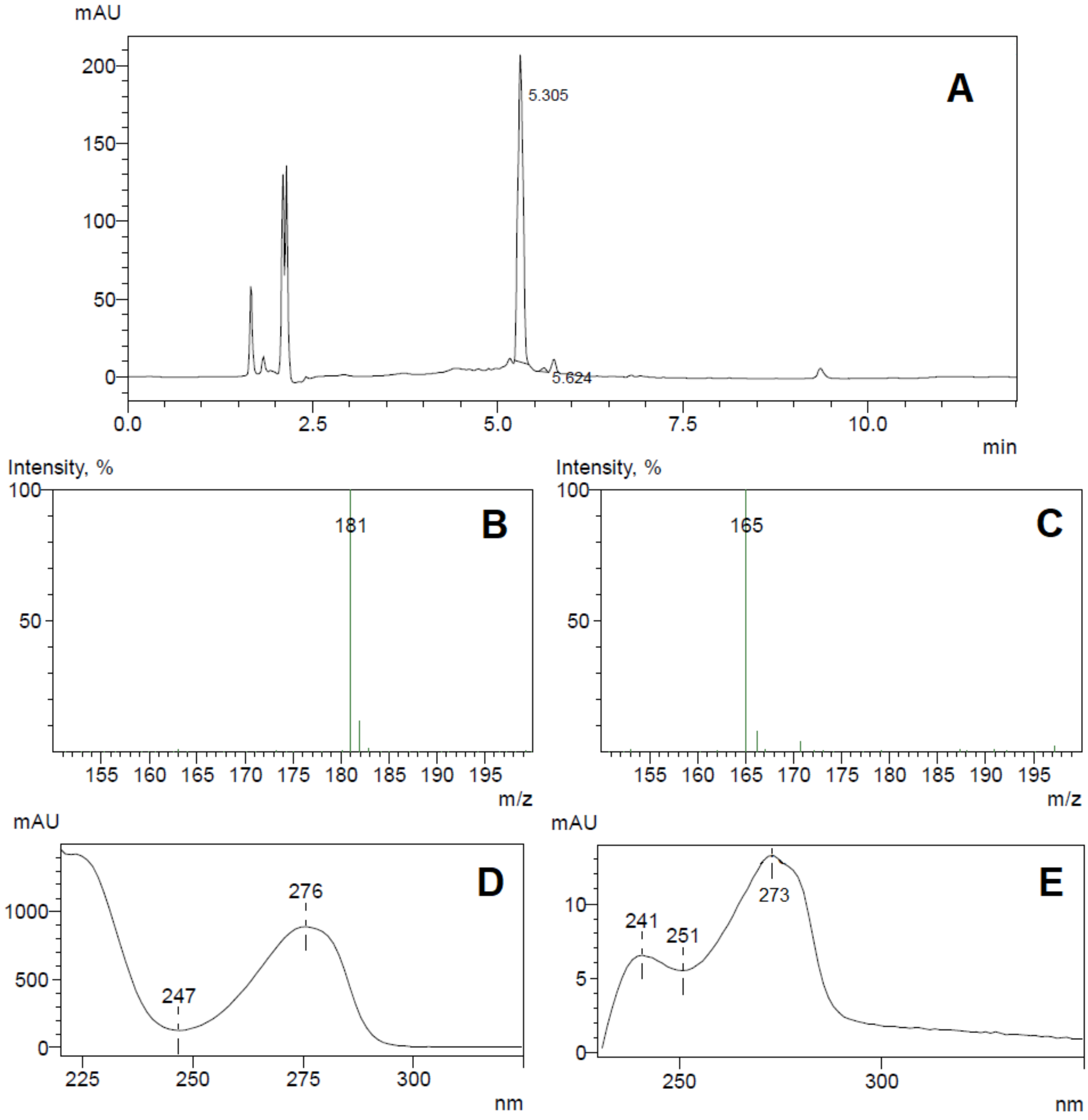

Figure S16. HPLC-MS analysis of 3-(2-hydroxyphenyl)-propionic acid bioconversion mixture. UV 254 nm trace of 3-(2-hydroxyphenyl)-propionic acid and its hydroxylated product 3-(2,3-dihydroxyphenyl)-propionic acid with retention times 5.624 min and 5.305, respectively (**A**). UV and MS spectra of peaks with retention times 5.305 min (**B**

and **D**) and 5.624 min (**C** and **E**). The negative ions $[M\text{-}H]^-$ generated are at m/z 181 (3-(2,3-dihydroxyphenyl)-propionic acid) and 161 (3-(2-hydroxyphenyl)-propionic acid).

REFERENCES

1. Godon, J.J.; Zumstein, E.; Dabert, P.; Habouzit, F.; Moletta, R. Molecular microbial diversity of an anaerobic digestor as determined by small-subunit rDNA sequence analysis. *Appl Environ Microbiol* **1997**, 63, 2802–2813.
2. Sivashanmugam, A.; Murray, V.; Cui, C.; Zhang, Y.; Wang, J.; Li, Q. Practical protocols for production of very high yields of recombinant proteins using *Escherichia coli*. *Protein Sci* **2009**, 18(5), 936–948.
3. Pan, Y.B.; Grisham, M.P.; Burner, D.M. A polymerase chain reaction protocol for the detection of *Xanthomonas albilineans*, the causal agent of sugarcane leaf scald disease. *Plant Dis* **1997**, 81, 189–194.
4. Gorelenkov, V.; Antipov, A.; Lejnine, S.; Darasella, N.; Yuryev, A. Set of novel tools for PCR primer design. *BioTechniques* **2001**, 31, 1326–1330.
5. Thompson, J.D.; Higgins, D.G.; Gibson, T.J. CLUSTAL-W improving the sensitivity of progressive multiple sequence alignment through sequence weighting, position-specific gap penalties and weight matrix choice. *Nucleic Acids Research* **1994**, 22, 4673–4680.
6. Tamura, K.; Peterson, D.; Peterson, N.; Stecher, G.; Nei, M.; Kumar, S. MEGA5: Molecular evolutionary genetics analysis using maximum likelihood, evolutionary distance, and maximum parsimony methods. *Molecular Biology and Evolution* **2011**, 28(10), 2731–2739.

7. Altschul, S.F.; Gish, W.; Miller, W.; Myers, E.W.; Lipman, D.J. Basic local alignment search tool. *J Mol Biol* **1990**, 215, 403–410.
8. Saitou, N.; Nei, M. The neighbor-joining method: a new method for reconstructing phylogenetic trees. *Molecular Biology and Evolution* **1987**, 4(4), 406–425.
9. Zackerkandl, E.; Pauling, L. Evolutionary divergence and convergence in proteins. *Evolving Genes and Proteins* **1965**, 97–166.
10. Babij, N.R.; McCusker, E.O.; Whiteker, G.T.; Canturk, B.; Choy, N.; Creemer, L.C.; De Amicis, C.V.; Hewlett, N.M.; Johnson, P.L.; Knobelsdorf, J.A.; Li, F.; Lorsbach, B.A.; Nugent, B.M.; Ryan, S.J.; Smith, M.R.; Yang, Q. NMR chemical shifts of trace impurities: Industrially preferred solvents used in process and green chemistry. *Org Process Res Dev* **2016**, 20, 661−667.
